# Prediction of upcoming global infection burden of influenza seasons after relaxation of public health and social measures during the COVID-19 pandemic: a modelling study

**DOI:** 10.1016/S2214-109X(22)00358-8

**Published:** 2022-10-11

**Authors:** Sheikh Taslim Ali, Yiu Chung Lau, Songwei Shan, Sukhyun Ryu, Zhanwei Du, Lin Wang, Xiao-Ke Xu, Dongxuan Chen, Jiaming Xiong, Jungyeon Tae, Tim K Tsang, Peng Wu, Eric H Y Lau, Benjamin J Cowling

**Affiliations:** a WHO Collaborating Centre for Infectious Disease Epidemiology and Control, School of Public Health, Li Ka Shing Faculty of Medicine, University of Hong Kong, Hong Kong Special Administrative Region, China; b Laboratory of Data Discovery for Health, Hong Kong Science Park, New Territories, Hong Kong Special Administrative Region, China; c Department of Preventive Medicine, Konyang University College of Medicine, Daejeon, South Korea; d Department of Genetics, University of Cambridge, Cambridge, UK; e College of Information and Communication Engineering, Dalian Minzu University, Dalian, China

## Abstract

**Background:**

The transmission dynamics of influenza were affected by public health and social measures (PHSMs) implemented globally since early 2020 to mitigate the COVID-19 pandemic. We aimed to assess the effect of COVID-19 PHSMs on the transmissibility of influenza viruses and to predict upcoming influenza epidemics.

**Methods:**

For this modelling study, we used surveillance data on influenza virus activity for 11 different locations and countries in 2017–22. We implemented a data-driven mechanistic predictive modelling framework to predict future influenza seasons on the basis of pre-COVID-19 dynamics and the effect of PHSMs during the COVID-19 pandemic. We simulated the potential excess burden of upcoming influenza epidemics in terms of fold rise in peak magnitude and epidemic size compared with pre-COVID-19 levels. We also examined how a proactive influenza vaccination programme could mitigate this effect.

**Findings:**

We estimated that COVID-19 PHSMs reduced influenza transmissibility by a maximum of 17·3% (95% CI 13·3–21·4) to 40·6% (35·2–45·9) and attack rate by 5·1% (1·5–7·2) to 24·8% (20·8–27·5) in the 2019–20 influenza season. We estimated a 10–60% increase in the population susceptibility for influenza, which might lead to a maximum of 1–5-fold rise in peak magnitude and 1–4-fold rise in epidemic size for the upcoming 2022–23 influenza season across locations, with a significantly higher fold rise in Singapore and Taiwan. The infection burden could be mitigated by additional proactive one-off influenza vaccination programmes.

**Interpretation:**

Our results suggest the potential for substantial increases in infection burden in upcoming influenza seasons across the globe. Strengthening influenza vaccination programmes is the best preventive measure to reduce the effect of influenza virus infections in the community.

**Funding:**

Health and Medical Research Fund, Hong Kong.

## Introduction

Since early 2020, various public health and social measures (PHSMs) have been implemented around the world to mitigate the COVID-19 pandemic caused by SARS-CoV-2.^1^, ^2^, ^3^, ^4^, ^5^, ^6^ These PHSMs also affected the transmission dynamics of directly transmitted viruses including the influenza virus.^1^, ^6^, ^7^, ^8^, ^9^ Several studies have reported evidence of a reduction in seasonal influenza activity in the 2019–20 season accounting for the direct and indirect impact of COVID-19 PHSMs,^1^, ^6^, ^8^, ^9^, ^10^ which also restricted influenza activity to low levels in successive seasons globally.^11^, ^12^ In fact, with low influenza circulation in the community for about 2 years, population immunity to influenza would have decreased substantially,^7^ although the seasonal influenza routine vaccination uptake increased during the COVID-19 pandemic in some countries.^13^ By mid-2022, COVID-19 PHSMs had been relaxed at various levels, either fully or partly, by the governments of different locations and countries.^14^, ^15^ Given the increases in susceptibility to influenza viruses along with the relaxation of COVID-19 PHSMs, the impact of upcoming influenza seasons could be considerably higher than that of the pre-COVID-19 pandemic seasons across the globe in terms of infections and related health-care seeking rates. An example of this is the very substantial winter influenza season in Australia that started in May, 2022.

Therefore, it is informative to predict the dynamics of future influenza seasons in different locations and countries and to assess the plausible scenarios of proactive intervention policies including increased influenza vaccination programme uptake to mitigate this excess infection burden. In this study, we aimed to first assess the effect of COVID-19 PHSMs on the transmissibility of influenza viruses, and then to predict the potential impact of forthcoming influenza seasons in several chosen locations and countries across the world.

**Research in context**

**Evidence before this study**

Public health and social measures (PHSMs) are important measures to control or mitigate epidemics and pandemics. We searched PubMed for published and peer-reviewed articles from Jan 1, 2020, to June 30, 2022, with the following search terms: (“PHSM” or “NPI”) AND (“SARS-CoV-2” or “COVID” or “COVID-19”) AND (“influenza” or “infectious disease”) AND (“forecast” or “future”). We found 57 studies assessing the effect of various PHSMs that were implemented to mitigate the COVID-19 pandemic. We found 19 studies forecasting upcoming epidemics, mainly for COVID-19, with only one recent country-based study in the USA predicting that the upcoming influenza seasons might increase upon the relaxation of COVID-19 PHSMs. These studies reported that COVID-19 PHSMs not only controlled COVID-19 transmission but also affected the transmission dynamics of several other directly transmitted respiratory viruses including the influenza virus and kept the community circulation of influenza close to zero across most parts of the globe during 2020 and 2021. Consequently, population immunity to influenza might have declined during the pandemic period.

**Added value of this study**

In this study, we used a mechanistic model allowing for waning immunity to estimate the potential intensity of upcoming influenza seasons globally after relaxation of COVID-19 PHSMs in geographically diverse countries and locations in both hemispheres. Along with assessing the effect of COVID-19 PHSMs, we predicted a potential 1–5-fold increase in peak magnitude and 1–4-fold increase in attack rate. Greater morbidity and mortality would result from the circulation of more antigenically distinct influenza strains in the upcoming season. An increase in vaccination uptake, perhaps along with re-imposition of some PHSMs, could mitigate the projected excess morbidity and mortality for these locations and countries.

**Implications of all the available evidence**

Once PHSMs are relaxed, given the declines in influenza immunity, there is certainly a risk of large upcoming influenza seasons. Forward planning for health-care management and public health policy should be able to mitigate this risk, by increasing vaccination uptake and by considering the re-implementation of some PHSMs to control influenza transmission.

## Methods

### Time series of influenza activity, COVID-19 PHSMs with data on influenza seasonal vaccination, and inter-border mobility

For this modelling study, we selected 11 geographically diverse countries and regions for analysis: mainland China, Hong Kong, Taiwan, South Korea, Singapore, Japan, Italy, Germany, the USA, the UK, and Australia. We retrieved data on weekly numbers of confirmed cases of influenza and total specimens tested, number of patient visits for influenza-like illness (ILI), and total consultations during 2017–21 from national sentinel and laboratory-based surveillance platforms in each location. We combined the influenza positivity rate and the ILI rate into an ILI+ proxy, a measure of influenza activity in the community, which has been shown to correlate with the incidence of influenza virus infections in the community.^16^, ^17^ The detailed derivation of the ILI+ proxy from the available information on these data streams with the data source for each country is described in appendix 1 (pp 3–6).

We retrieved information on timing and duration of PHSMs implemented in response to the COVID-19 pandemic as reported by governments, agencies, published reports, research articles, and news for different locations and countries in our study (appendix 1 p 33, appendix 2). We first classified these PHSMs into case-based, community-wide, and travel-related control measures with their implementation timing and duration for each location and country (appendix 1 p 7). We also retrieved the information on timing, duration, and coverage of seasonal routine influenza vaccination programmes from the available government and media reports and literature in the studied locations and countries during the study period (appendix 1 pp 33–37). We calculated the respective routine vaccination rates, τ_r_, for each location and country across the study period. We compiled information on yearly number of visits (inter-border population mobility) in each location and country during 2017–22 from reports or websites available from respective governments and travel agencies (appendix 1 p 38). The weekly infection seeding, ɛ*(t)*, was evaluated from the travel data and explored through a sensitivity analysis (appendix 1 pp 7–8). The study protocol was approved by the Institutional Review Board of the University of Hong Kong (UW 21–775).

### Impact assessment of COVID-19 PHSMs on influenza transmissibility

We used the methods proposed by Cori and colleagues^18^ to estimate transmissibility through the effective reproduction number, *R*_t_, which represents the average number of further infections that result from a case infected at time *t* (appendix 1 pp 8–10). To evaluate the impact of COVID-19 PHSMs, we compared the transmissibility before and during the intervention period for each location. The changes in mean *R*_t_ values indicate the impact of COVID-19 interventions on seasonal influenza transmissibility as the immediate effect. To assess the overall effect of interventions, we used a regression framework on *R*_t_^19^ and constructed a log-linear multivariable regression model accounting for the depletion of susceptibles and the timing and intensity of the interventions at time *t*. The detailed derivation of the regression model and evaluation of overall changes in transmissibility are provided in appendix 1 (pp 8–10).

### Impact assessment of COVID-19 PHSMs on influenza attack rate

In theory, the measure of transmissibility *R*_t_ is potentially driven by depletion of susceptibles, *h*_t_, along with extrinsic factors including the stringency of COVID-19 PHSMs, *C*_t_. We first evaluated the transmission rate, β_t_, as another measure of transmissibility that is technically free from the effect of depletion of susceptibles, and hence a function of the initial transmission rate, β_0_, and *C*_t_ (ie, β_t_= β_0_ × e^λCt^), assuming other factors were constant during the short period of time. Then, using β_t_, we simulated influenza activity by constructing the standard susceptible–exposed–infected–recovered transmission model under the counterfactual scenario of implementation timing of COVID-19 PHSMs. We did simulations to predict the incidence under no effect of PHSMs, by setting λ=0, and we compared the attack rates, simulated under the estimated λ (in the regression model). We extended this modelling framework to address the sustained zero (or near-zero, except for some sporadic outbreaks) cases of influenza viruses globally during the COVID-19 pandemic. The detailed simulation framework is provided in appendix 1 (pp 10–11).

### Prediction of the next influenza seasons

We developed a simulation-based predictive model framework to infer what might happen in upcoming influenza seasons after the relaxation of COVID-19 PHSMs. We first fitted a stochastic susceptible–vaccinated–infectious–recovered–susceptible model (appendix 1 pp 20, 39) to the ILI+ proxy from Oct 1, 2017, to Jan 31, 2020, for these locations and countries to estimate influenza dynamics under routine vaccination programmes before PHSMs became effective in February, 2020 (appendix 1 pp 12–19). We used partly observed Markov process models, the state space models embedded with filtering techniques by the R package pomp.^20^ The model parameters were estimated by the iterated filtering method (IF2). Parameters were searched with a grid of a sufficient number of random combinations of the samples by Latin hypercube sampling technique to obtain the estimates with global maximum likelihood. However, because the IF2 algorithm might not always return the estimates with maximum likelihood,^20^ we also applied the Monte Carlo-adjusted profile algorithm to the likelihood profile to update the estimates and obtained the corresponding 95% CIs (appendix 1 pp 12–19).^21^

Using the parameters estimated by the predictive models, we simulated future influenza seasons in the selected locations under different effects of COVID-19 PHSMs on influenza viruses. We assumed that the COVID-19 PHSMs would reduce the time-varying reproduction number, *R*_e_(*t*), for influenza to a certain level, denoted as *R*_e_^reduced^(*t*), such that the influenza activity was kept undetectable since February, 2020. On relaxation of these COVID-19 PHSMs, *R*_e_(*t*) would again recover up to some proportion of *R*_e_(*t*) at time *t*, at a level denoted as *R*_e_^recovered^(*t*) for the next seasons. Therefore, the magnitude of future influenza seasons primarily depends on the effective duration, υ, of COVID-19 PHSMs and the proportion of *R*_e_(*t*) that has recovered, *p*^recovered^, considering different degrees of PHSMs relaxation (appendix 1 pp 17–19). Moreover, travel-related infectious seeding visits after PHSMs relaxation were considered in the model, assuming 0·1% (0·02% and 0·5% for sensitivity) of travellers were infectious. We predicted future seasons when the PHSMs might be relaxed in October, 2021, April, 2022, and October, 2022, and allowed the model to predict the subsequent seasons until 2023 under the routine influenza vaccination programme (with same rate and timing) for these locations studied. We estimated the excess burden in terms of infections and peak activity for future epidemics by measuring the δ-fold rise in peak magnitude and epidemic size, comparing them with baseline estimates during the 2017–19 seasons before the COVID-19 pandemic (appendix 1 pp 12–19).

Finally, apart from routine vaccination programmes, we used this framework to estimate the possible contribution of enhanced or one-off additional influenza vaccination programmes in advance of the start of the following influenza season, which could mitigate the risk of larger outbreaks (appendix 1 pp 18–19). We explored the proportion of additional vaccination coverage by accounting for the vaccination duration, τ_o_^L^, and the vaccination rate, τ_o_, and we compared the predicted upcoming seasons with and without vaccination, assuming that R_e_(*t*) was fully recovered (ie, *p*^recovered^=1) after the relaxation of COVID-19 PHSMs. Setting the peak influenza activity during the 2017–19 seasons as the reference-level activity, we evaluated the fold rise of predicted upcoming seasons and assessed the optimum τ_o_^L^ and τ_o_ to reduce excess infection burden (ie, to make sure that the δ-fold rise in peak magnitude equals 1).

### Role of the funding source

The funders of the study had no role in study design, data collection, implementation of the study, data analysis, or reporting of the results.

## Results

**Figure 1:**
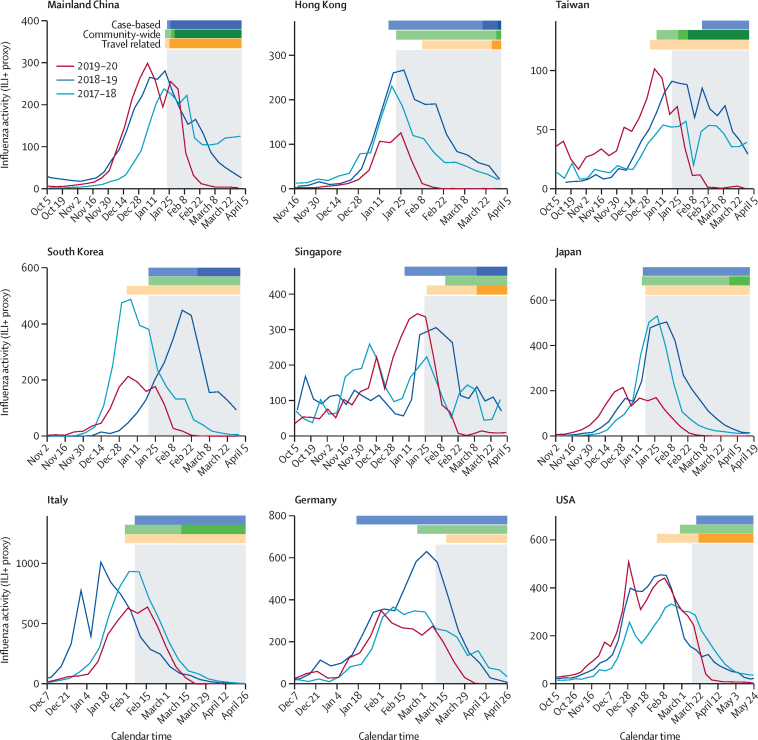
Reduction in influenza activity (weekly ILI+ proxy) in nine different locations and countries across the globe during the 2019–20 season by COVID-19 PHSMs in 2020 Time series of ILI+ proxy for the 2019–20, 2018–19, and 2017–18 seasons in these locations. The timing and the magnitude indicators of PHSMs are classified as case-based, community-wide, or travel related. The grey shaded regions indicate the timing of the COVID-19 pandemic for each location, identified for the effective impact of COVID-19 PHSMs on influenza as the indicator of pre-pandemic and pandemic scenarios. The UK and Australia are not shown because the COVID-19 pandemic started after their respective influenza seasons had concluded. ILI+ proxy=combination of influenza positivity rate and influenza-like illness rate. PHSMs=public health and social measures.

We observed that influenza activity in each location substantially declined at various levels in the 2019–20 season during the early COVID-19 pandemic (figure 1). For most locations, COVID-19 PHSMs were in place around the peak or during the post-peak of the 2019–20 seasonal influenza epidemics, when a range of PHSMs were implemented simultaneously with varied intensity over time (figure 1, appendix 1 pp 21, 33).

**Figure 2:**
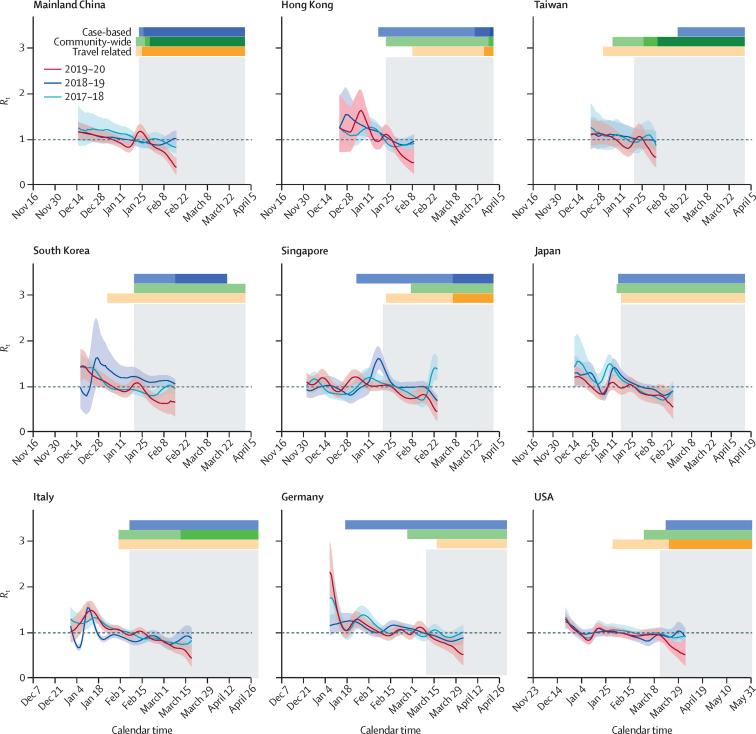
Reduction in influenza transmissibility (R_t_) in nine different locations and countries across the globe during the 2019–20 season by COVID-19 PHSMs in 2020 The solid lines represent the weekly real-time estimates of *R*_t_ for the 2019–20, 2018–19, and 2017–18 seasons in these locations. Shaded regions represent the respective 95% CIs. The dotted lines indicate the transmission threshold (*R*_t_=1). The timing and the magnitude indicators of PHSMs are classified as case-based, community-wide, or travel related. The grey shaded regions indicate the timing of the COVID-19 pandemic for each location, identified for the effective impact of COVID-19 PHSMs on influenza as the indicator of pre-pandemic and pandemic scenarios. The UK and Australia are not shown because the COVID-19 pandemic started after their respective influenza seasons had concluded. PHSMs=public health and social measures. *R*_t_=time-varying reproduction number.

The immediate reductions in transmissibility accounting for the effect of PHSMs were highly significant (p values <0·001) and with estimated reductions ranging from 17% (95% CI 13 to 21) to 41% (35 to 46) across the locations and countries, with comparatively higher reductions for Hong Kong and Italy, for which the COVID-19 PHSMs were implemented just before or around the expected seasonal peak (figure 2, table, appendix 1 p 21). The estimates of coefficients, λ, of overall reduction in transmissibility ranged from –0·13 (–0·15 to –0·10) to –0·04 (–0·05 to –0·03), corresponding to significant reductions in *R*_t_ in a similar range, from 14·5% (8·0 to 20·4) to 31·1% (26·1 to 35·6), across locations and countries (table).

**Table:** Estimates of reduction in transmissibility and attack rate of the influenza virus in selected locations and countries during 2020

	**Reduction in transmissibility (R_t_ analysis)**		**Reduction in transmissibility (regression analysis)**		**Reduction in infection or attack rate (simulation analysis)**	
	Reduction in R_t_, %	p value (best length)	λ	Reduction, %	Reduction in infection, %	Timing of PHSMs (weeks from peak)
Mainland China	19·56% (14·37 to 24·75)	<0·0001 (27)	−0·13 (−0·18 to −0·09)	29·98% (19·60 to 38·66)	10·26% (2·86 to 14·20)	2
Hong Kong	40·55% (35·19 to 45·91)	<0·0001 (19)	−0·06 (−0·12 to −0·01)	16·46% (0·05 to 29·79)	21·02% (10·66 to 26·65)	0
Taiwan	17·34% (13·30 to 21·38)	0·0006 (26)	−0·04 (−0·06 to −0·02)	19·29% (10·99 to 26·58)	14·31% (11·70 to 16·11)	2
South Korea	25·89% (22·53 to 29·24)	<0·0001 (27)	−0·02 (−0·07 to 0·03)	4·95%* (−7·63 to 16·00)	5·08% (1·45 to 7·21)	1
Singapore	23·02% (21·27 to 24·76)	<0·0001 (17)	−0·04 (−0·05 to −0·03)	20·76% (17·32 to 24·02)	24·80% (20·82 to 27·51)	1
Japan	20·67% (17·35 to 23·99)	<0·0001 (39)	−0·12 (−0·17 to −0·06)	14·47% (7·99 to 20·41)	7·57% (3·72 to 10·34)	1
Italy	33·19% (28·96 to 37·42)	<0·0001 (28)	−0·09 (−0·14 to −0·05)	23·24% (19·96 to 26·32)	13·66% (12·46 to 14·70)	−1
Germany	26·75% (20·60 to 32·90)	0·0003 (21)	−0·13 (−0·15 to −0·10)	29·40% (24·95 to 33·53)	10·96% (10·10 to 11·61)	3
USA	20·41% (17·39 to 23·43)	<0·0001 (24)	−0·10 (−0·12 to −0·09)	31·06% (26·13 to 35·62)	7·71% (7·50 to 7·87)	5

Data are % (95% CI) or estimates of coefficients (95% CI), unless otherwise specified. The percentages of reduction were calculated in different analyses. These analyses were not done for the UK and Australia, because the COVID-19 pandemic started after their respective influenza seasons had concluded. λ=estimate of coefficient. PHSMs=public health and social measures. Rt=effective reproduction number.

* For South Korea, the regression analysis didn't return a significant reduction.

**Figure 3:**
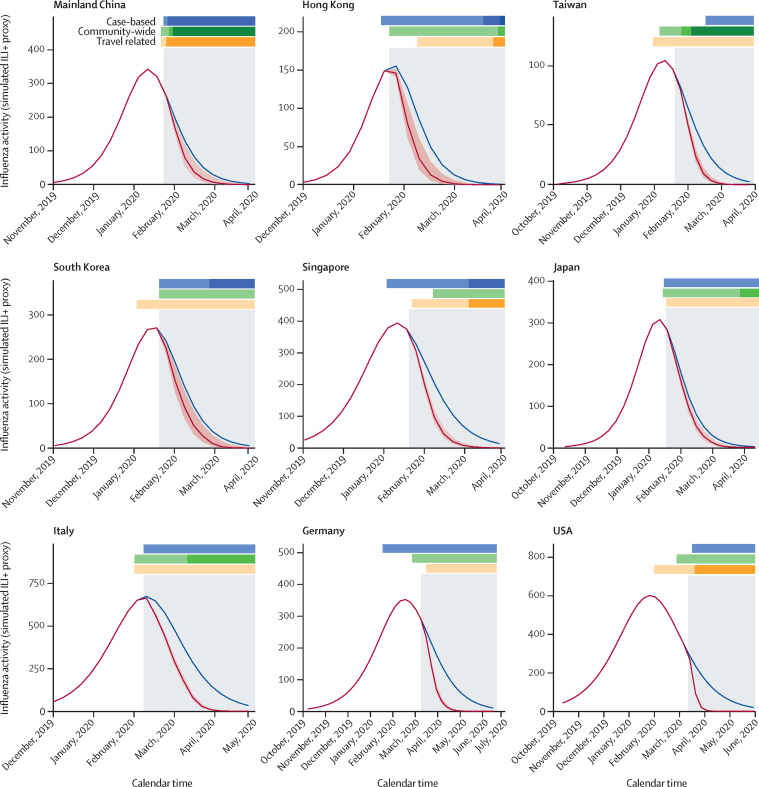
Reduction in influenza attack rates in nine different locations and countries across the globe during the 2019–20 season by COVID-19 PHSMs in 2020 Simulated incidence of influenza viruses with and without implementation of COVID-19 PHSMs. Blue lines indicate the number of cases under the scenario of no COVID-19 PHSMs; red lines indicate the number of cases with the effect of COVID-19 PHSMs in 2020, with 95% CIs in red shaded regions. The difference between blue and red lines represents the respective reduction in incidence of infections for each location. The timing and the magnitude indicators of PHSMs are classified as case-based, community-wide, or travel-related. The grey shaded regions indicate the timing of the COVID-19 pandemic for each location, identified for the effective impact of COVID-19 PHSMs on influenza as the indicator of pre-pandemic and pandemic scenarios. The UK and Australia are not shown because the COVID-19 pandemic started after their respective influenza seasons had concluded. ILI+ proxy=combination of influenza positivity rate and influenza-like illness rate. PHSMs=public health and social measures.

Under the simulated framework with counterfactual realisation of the effect of COVID-19 PHSMs in the community, the reductions in influenza attack rates (cumulative incidence) were estimated to be from 5·1% (1·5–7·2) to 24·8% (20·8–27·5) for locations and countries with no pre-existing population immunity assumption (figure 3, table, appendix 1 p 22). In sensitivity analyses considering different levels of possible pre-existing population immunity (up to 30%) for influenza at the start of the epidemic, we found the reductions in influenza attack rates to be similar across different levels of pre-existing immunity, but slightly smaller in the population with no pre-existing immunity (appendix 1 p 40). The coefficient, λ_min_, as the optimal effect of PHSMs to contain influenza circulation during the 2020 season (summer to winter) and successive seasons, was estimated to be –0·2 with seeding (ι=10 per day), and λ_min_ became much larger as ι increased (appendix 1 p 23).

**Figure 4:**
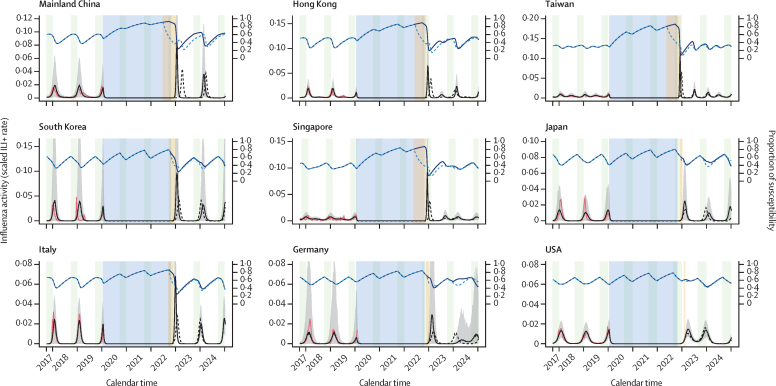
Prediction of upcoming influenza activity when COVID-19 PHSMs are relaxed in October, 2022, (before the winter season) with proactive vaccination programmes (one-off vaccination timing, duration, and rate) to mitigate the excess influenza activity in nine locations and countries The forecast of upcoming seasons was evaluated on the basis of the predictive model framework by fitting the susceptible–vaccinated–infected–recovered–susceptible models on influenza activity (observed incidence) data from October, 2017, to January, 2020, and accounting for increased susceptibility and the reduced effect of COVID-19 PHSMs on influenza by location and country. Red lines represent the observed incidence rate, and blue shaded regions indicate the period under COVID-19 PHSMs for each location. Light green shaded regions indicate the routine vaccination periods for each location. Solid black lines represent the mean forecast (in 1000 simulations) of the incidence rate for the 2022 winter season under no proactive vaccination or intervention for influenza, with grey shaded areas representing 2·5–97·5th quantiles. Dashed black lines represent the mean forecast of infection rate under a one-off vaccination programme to mitigate the excess infection burden in the first upcoming season of influenza. The vaccination start timing was set to the day when the incidence rate crossed a predefined threshold, and the vaccination period (yellow-shaded area) was optimised to achieve baseline activity (as was in the 2017–19 seasons) with a projected vaccination rate of 0·05 per week of the total susceptible population. Blue lines represent the proportion of the susceptible population with (solid line) and without (dashed line) vaccination. We did this prediction for successive seasons until December, 2024. The UK and Australia are not shown because the COVID-19 pandemic started after their respective influenza seasons had concluded. ILI+=combination of influenza positivity rate and influenza-like illness rate. PHSMs=public health and social measures.

**Figure 5:**
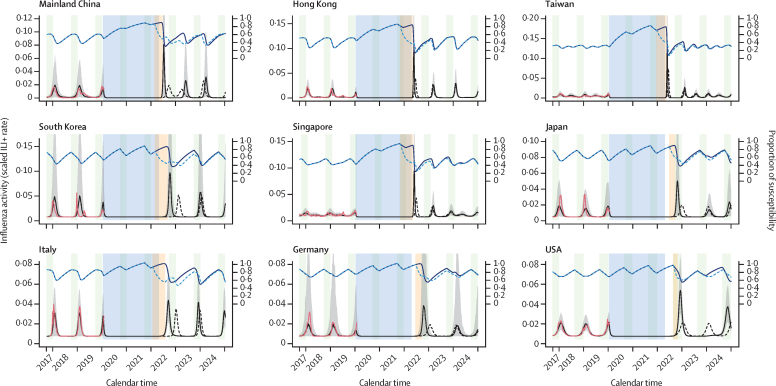
Prediction of ongoing influenza activity when COVID-19 PHSMs are relaxed in April, 2022, (before the summer season) with proactive vaccination programmes (one-off vaccination timing, duration, and rate) to mitigate the excess influenza activity in nine locations and countries The forecast of upcoming seasons was evaluated on the basis of the predictive model framework by fitting the susceptible–vaccinated–infected–recovered–susceptible models on influenza activity (observed incidence) data from October, 2017, to January, 2020, and accounting for increased susceptibility and the reduced effect of COVID-19 PHSMs on influenza by location and country. Red lines represent the observed incidence rate, and blue shaded regions indicate the period under COVID-19 PHSMs for each location. Light green shaded regions indicate the routine vaccination periods for each location. Solid black lines represent the mean forecast (in 1000 simulations) of the incidence rate for the 2022 summer season under no proactive vaccination or intervention for influenza, with grey shaded areas representing 2·5–97·5th quantiles. Dashed black lines represent the mean forecast of infection rate under a one-off vaccination programme to mitigate the excess infection burden in the first upcoming season of influenza. The vaccination start timing was set to the day when the incidence rate crossed a predefined threshold and the vaccination period (yellow shaded area) was optimised to achieve baseline activity (as was in the 2017–19 seasons) with a projected vaccination rate of 0·05 per week of the total susceptible population. Blue lines represent the proportion of the susceptible population with (solid line) and without (dashed line) vaccination. We did this prediction for successive seasons until December, 2024. The UK and Australia are not shown because the COVID-19 pandemic started after their respective influenza seasons had concluded. ILI+ proxy=combination of influenza positivity rate and influenza-like illness rate. PHSMs=public health and social measures.

We estimated the proportions of susceptibility to be in the range of 0·31–0·69 (with lower proportions for Taiwan and Singapore) during the pre-COVID-19 pandemic period and to increase up to the maximum range (about 0·80–0·90) after about 2 years of no sustained circulation of influenza in these locations. Assuming that PHSMs against COVID-19 relax completely in October, 2022, ensuring that *R*_e_*(t)* values fully recover to their pre-COVID-19 pandemic levels for the 2022–23 season, we estimated the δ-fold rise in peak magnitude to range from 1·1 (2·5–97·5th percentile 1·0–1·2) to 4·8 (4·8–4·9) for most locations, except Taiwan (13·6, 12·9–14·3) and Singapore (11·7, 11·0–12·2), and the δ-fold rise in epidemic size to range from 1·1 (1·0–1·2) to 3·2 (3·1–3·3) for most locations, with higher δ-fold rise in epidemic size for Taiwan and Singapore (figure 4, appendix 1 p 42). We estimated similar δ-fold rises for the ongoing 2022 summer season (figure 5) assuming full relaxation of COVID-19 PHSMs in April, 2022, and for the retrospective 2021–22 winter season in the northern hemisphere (appendix 1 p 24) assuming full relaxation of COVID-19 PHSMs in October, 2021. We found the δ-fold rises in peak magnitude and epidemic size for winter seasons to be slightly smaller compared with those of summer seasons in tropical and subtropical locations in the northern hemisphere (appendix 1 p 42). As an extension of this framework, the predictions for southern and northern mainland China, the UK, and Australia were found to be similar for the upcoming 2022–23 season (appendix 1 pp 25, 42).

Allowing for various degrees of relaxation of COVID-19 PHSMs over time, we found a positive association between *p*^recovered^ and the metrics of δ-fold rise in peak magnitude and epidemic size (appendix 1 pp 26–27). We found that the δ-fold rise in peak magnitude would reach close to 1 when *p*^recovered^ was about 0·3–0·8 for most of the locations across the seasons, except Taiwan (approximately 0·2), Singapore (approximately 0·3), and Australia and winter seasons for the USA and Japan (approximately 0·9–1·0; appendix 1 p 26). We found similar results for the δ-fold rise in epidemic size for these locations (appendix 1 p 27). Any values of *p*^recovered^ greater than these estimates would return different estimates of δ-fold rise (δ>1) in peak magnitude and epidemic size (unless multiple peaks occurred within a year). The peak timing of the first predicted upcoming influenza seasons from the timing of the relaxation of COVID-19 PHSMs were negatively (non-linear) associated with *p*^recovered^ across locations and upcoming seasons (appendix 1 p 28). With 50% recovery of *R*_e_*(t)* (ie, *p*^recovered^=0·5), the median peak timing delay could be about 11·9 weeks (IQR 4·5–16·6) for the 2022 season and about 7·0 weeks (5·0–9·5) for the 2022–23 season.

We found that an additional maximum of up to 30% of the total susceptible population would need to be vaccinated with a one-off vaccination programme (for <3 months with a vaccination rate of 0·05 per week) in most locations to alleviate the excess burden of peak activity for the 2022–23 influenza season, except in southern mainland China, Hong Kong, Taiwan, and Singapore (figure 4, appendix 1 pp 29, 31).

However, for subtropical locations and countries such as southern mainland China, Hong Kong, Taiwan, and Singapore, which had higher δ-fold rise in peak magnitude, the alleviation of the excess burden of peak activity would require either vaccination with a longer period (>26 weeks), a higher vaccination rate (rate of >0·05 per week in the total susceptible population), or both. Additionally, we found that a higher vaccination rate during a longer period (ie, one-off vaccination programmes starting too early) might lead to a resurgence of the influenza epidemics (ie, δ-fold rise >1 for peak magnitude and epidemic size), accounting for delayed peaks in temperate locations including South Korea, Japan, Italy, Germany, the USA, and the UK (appendix 1 pp 29–30). The travel-related infection seeding after relaxation of COVID-19 PHSMs had a similar effect on the δ-fold rise in peak magnitude with different percentages of seeding (0·02–0·5%; appendix 1 p 32).

## Discussion

We found that PHSMs for COVID-19 significantly reduced influenza transmission globally during 2020. The effect of COVID-19 PHSMs in decreasing the influenza activity has been reported for different locations and countries in the northern hemisphere for winter influenza seasons,^1^, ^6^, ^7^, ^9^, ^10^, ^22^, ^23^, ^24^ and in the southern hemisphere for summer influenza seasons.^8^, ^25^, ^26^ We identified the changes in transmissibility of the influenza virus in these locations and evaluated the immediate reduction in transmissibility (up to 41%) after the implementation of PHSMs in response to COVID-19 during early 2020. The reductions varied across the locations on the basis of timing and intensity of COVID-19 PHSMs and were found to have higher impact when the timing of PHSMs coincided with the peak activity of influenza seasons. The overall reduction in transmissibility could have led to up to a 25% reduction in attack rate, avoiding infections in the community during the 2019–20 influenza season. Previous studies have reported a similar effect of COVID-19 PHSMs in influenza activity and transmissibility during the 2019–20 season in several locations,^1^, ^7^ some with even higher reduction in infections, up to 60% for the USA,^6^ when estimated for a longer period (up to 10 weeks). The effect of COVID-19 PHSMs was not only restricted in the 2019–20 influenza season, but also extended beyond 2020, with no sustained influenza activity in the community across the globe during the COVID-19 pandemic. This ensured the potential of non-pharmaceutical interventions (eg, PHSMs) to keep influenza activity at near-zero for almost 2 years. Along with influenza, the dynamics of other infections were also modulated by COVID-19 PHSMs in early 2020.^8^, ^10^, ^22^, ^24^, ^25^

Our model-based prediction suggests a maximum of 1–5-fold rise in peak magnitude and 1–4-fold rise in epidemic size for the upcoming influenza 2022–23 winter season across the studied locations and countries in the northern hemisphere, with a much higher fold rise expected in Singapore and Taiwan. The fold rise in peak magnitude and epidemic size varied across the locations, accounting for the degree of recovery in transmissibility *R*_e_*(t)* driven by the various levels of relaxation of COVID-19 PHSMs and the increase in population susceptibility from pre-pandemic levels. We estimated that the pre-pandemic population susceptibility was lower than in other locations, with about 30–50% of the total population for Singapore and Taiwan, which led to a significantly higher fold rise in peak magnitude and epidemic size for the upcoming influenza seasons in these locations. Additionally, higher vaccination coverage in the past few years, particularly for target groups (over 24% coverage), in Taiwan^27^ and Singapore,^28^ with year-round influenza activity,^29^, ^30^ might help to maintain a higher population immunity than in other locations, which might lead to the lower estimates of population susceptibility before the COVID-19 pandemic. Conversely, strong seasonality in temperate locations could allow for the regular recovery of infections during the pre-COVID-19 pandemic period, resulting in a reduced surge of susceptibility in the population during the COVID-19 pandemic. However, several countries have already started relaxing COVID-19 PHSMs and opting for living with COVID-19 policies,^15^ under which the transmissibility *R*_e_*(t)* of influenza is likely to approach or even exceed its pre-pandemic values. Therefore, the δ-fold rise in peak magnitude and epidemic size in these locations and countries might be modulated by these factors accordingly, as presented in the counter-factual-based sensitivity analysis with recovery of *R*_e_*(t)*, although travel-based infection seeding after COVID-19 PHSM relaxation is less sensitive to the outcomes. Additionally, one-off proactive vaccination might be able to prevent the excess infection burden of upcoming influenza seasons in temperate locations, but might not be sufficient for subtropical locations such as southern mainland China, Hong Kong, Taiwan, and Singapore, where some effective PHSMs could be considered to mitigate the excess burden.

Our estimates showed that the δ-fold rises in epidemic size within 1 year after PHSM relaxation across the locations are more consistent than those for peak magnitude. Therefore, the prediction of peak timing in advance is crucial and was found to be negatively associated with the level of PHSM relaxation (which leads to *R*_e_(*t*) recovery). Peak timings for upcoming influenza seasons in locations and countries in tropical and subtropical latitudes are expected to appear earlier than those of temperate locations, which could be driven by other climatic and environmental factors, especially in the summer seasons in Hong Kong, mainland China, Taiwan, and Singapore.^30^, ^31^ Our findings recommend that preparations be made for proactive intervention policies to mitigate the excess infections or health burden for upcoming influenza seasons.

Our study has some limitations. First, the framework did not disentangle the effect of individual PHSMs but rather considered the combined effects of these interventions on predictive model construction, as they were implemented almost simultaneously. Second, the predictive models were based on the ILI+ rate (proxy), which was not uniform across locations and might be affected by issues of ascertainment and health-seeking behaviour during the COVID-19 pandemic in 2020. Third, we could not retrieve age-specific influenza data in these locations, which could provide more insight on future transmission patterns and provide an assessment of age-stratified, target-based, and setting-based proactive intervention and vaccination strategies. Fourth, our prediction model accounted for the effect of easing COVID-19 PHSMs and increased susceptibility to seasonal influenza viruses in the population due to waning immunity from natural infections, expecting the possible usual seasonal antigenic drift (even a tiny change) in the virus.^6^, ^7^, ^32^ However, antibody titre-based studies reported uncertainty in such waning immunity for the same influenza viruses and the degree of antigenic changes (drift or shift) in influenza viruses with the near-absence of seasonal influenza during the COVID-19 pandemic.^33^, ^34^ This uncertainty could affect our prediction of future influenza infection burden. Finally, we did not explore the possibility of vaccine effectiveness (or mismatches) and any cross-protection of COVID-19 infection on influenza virus infections,^35^ which could affect our forecasting of the burden of upcoming influenza seasons.

In conclusion, our results warn of the potential for a substantial infection burden for upcoming influenza seasons. Timely influenza vaccination programmes could be the best preventive measure to reduce the impact of influenza in the community. A combination of PHSMs, targeted vaccination programmes (in children and older adults), school-based vaccination programme promotion, and joint vaccination schemes for COVID-19 and influenza could be implemented before the upcoming seasons to mitigate the combined burden of COVID-19 and influenza.

## Data sharing

The data on physical distancing policies are contained in appendix 2 and are publicly available at https://osf.io/9ve5j/ (or https://doi.org/10.6084/m9.figshare.18517028).

## Declaration of interests

BJC reports honoraria from AstraZeneca, Fosun Pharma, GSK, Moderna, Pfizer, Roche, and Sanofi. All other authors declare no competing interests.
