# Supplementary material for: Prediction of upcoming global infection burden of influenza seasons after relaxation of public health and social measures during the COVID-19 pandemic: a modelling study
Source: Lancet Glob Health. 2022 Oct 11;10(11):e1612–22. doi: 10.1016/S2214-109X(22)00358-8 (PMC9573849; doi:10.1016/S2214-109X(22)00358-8)
Supplement: Supplementary appendix 1 [file mmc1.pdf]

THE LANCET
Global Health

Supplementary appendix 1

This appendix formed part of the original submission and has been peer reviewed. We post it as supplied by the authors.

Supplement to: Ali ST, Lau YC, Shan S, et al. Prediction of upcoming global infection burden of influenza seasons after relaxation of public health and social measures during the COVID-19 pandemic: a modelling study. *Lancet Glob Health* 2022;
10: e1612–22.

Appendix for

Prediction of upcoming global infection burden of influenza seasons after relaxation of public health and social measures during the COVID-19 pandemic: a modelling study

Sheikh Taslim Ali[1,2,†], Yiu Chung Lau[1,2, †], Songwei Shan[1,2,†], Sukhyun Ryu[3,†], Zhanwei Du[1,2], Lin Wang[4], Xiao-Ke Xu[5], Dongxuan Chen[1,2], Jiaming Xiong[1,2], Jungyeon Tae[3], Tim K. Tsang[1], Peng Wu[1,2], Eric H. Y. Lau[1,2], Benjamin J. Cowling[1,2,*]

Affiliation:

[1] WHO Collaborating Centre for Infectious Disease Epidemiology and Control, School of Public Health, Li Ka Shing Faculty of Medicine, The University of Hong Kong, Hong Kong Special Administrative Region, China

[2] Laboratory of Data Discovery for Health Limited, Hong Kong Science Park, New Territories, Hong Kong.

[3] Department of Preventive Medicine, Konyang University College of Medicine, Daejeon 35365, Republic of Korea.

[4] Department of Genetics, University of Cambridge, Cambridge CB2 3EH, UK.

[5] College of Information and Communication Engineering, Dalian Minzu University, Dalian 116600, China.

[†] Contributed equally.

Corresponding author:

* Prof. Benjamin J Cowling, School of Public Health, Li Ka Shing Faculty of Medicine, The University of Hong Kong, 7 Sassoon Road, Pokfulam, Hong Kong.

Tel: +852 3917 6711; Fax: +852 3520 1945; email: bcowling@hku.hk

Contents:

Sl. No.	Details	Page No.
1	Sources of influenza data and calculation of influenza activity (ILI+ proxy)	03
2	Collection of location- or country-wise data on PHSMs for COVID-19	07
3	Collection of data on sessional routine vaccination (timing, duration and coverage) and yearly number of visitors for the studied countries/ locations.	07
4	Log-linear regression model for transmissibility, R_t	08
5	Susceptible-Exposed-Infected-Recovered (SEIR) transmission model and simulation of the attack rates	10
6	Predictive mechanistic model to infer next influenza seasons	12-19
6.1	Model construction and specification	12
6.2	Likelihood and parameter estimations	15
6.3	Data rescaling	17
6.4	Model fitting for Northern and Southern Mainland China	17
6.5	Predictive simulation for upcoming flu seasons	17
6.6	Assessment of excess burden for upcoming seasons	19
6	Figures S1 to S13	20-32
7	Tables S1 to S7	33-42
8	References	43

1. Sources of influenza data and calculation of influenza activity (ILI+ rate proxy)

We retrieved the data on influenza activity in 11 diverse locations (countries or regions) in the globe, including mainland China, Hong Kong, Taiwan, South Korea, Singapore, Japan, Italy, Germany, the United States of America (USA), the United Kingdom (UK) and Australia during 2017 to 2021. We considered these countries based on the data availability and timing of the 2019-2020 season coincided with the public health and social measures (PHSMs) for COVID-19 pandemic.

We first retrieved the time series on weekly number of influenza tested positive (φ_p) and total number of specimens tested (φ_T) to evaluate the influenza positive rate (φ_p/φ_T). However, the φ_p/φ_T suffers from the reporting rate and the capacity of effective surveillance and testing system of that country or location, hence unable to fully represent the influenza activity in the community. Therefore, we further retrieved the time series on weekly number of ILI consultations (ψ_{ILI}) and total number of consultations/ visits to outpatient clinics or GPs (ψ_T) to calculate the ILI consultation rate (ψ_{ILI}/ψ_T) in the community. This possibly covers a wider spectrum of community influenza cases, despite lower diagnostic specificity. We then incorporated these different but comparable information from two available sources to quantify the community-level influenza activities as ILI+ rate by multiplying influenza positive rate (φ_p/φ_T) with ILI consultation rate (ψ_{ILI}/ψ_T). Further, we rescaled the ILI+ rate according to the reporting information by the sentinel system of that location and finally evaluated the representative influenza activity as $\text{ILI} + \text{Proxy} = \varphi_p/\varphi_T \times \psi_{ILI}/\psi_T \times c,\ c$ (≥ 1) is rescaling factor. For example, in Hong Kong we have 60 out of over 600 GPs are under central surveillance network, therefore simply the value of c will be ~10 [1]. We have previously shown that the ILI+ proxy for influenza A(H1N1)pdm09 was a close linear correlate of the incidence of hospitalisations for H1N1pdm09 in Hong Kong [2], and it has been argued that this ILI+ proxy metric should be a better correlate of incidence than ILI rates or laboratory detection rates alone [3]. This proxy measure was used in several studies to minimize underreporting bias in the laboratory-confirmed influenza data and to address unobservability of influenza virus infections in the community [1,4-8]. Noticeably, there were variations in the surveillance systems across

the locations, some countries/regions including mainland China (reported by Southern mainland China and Northern mainland China separately), Hong Kong, Taiwan, South Korea and the United States reported the ILI consultation rate (ψ_{ILI}/ψ_T), while others didn't report the data on consolations directly and hence the ILI consultation rates were adjusted with available information.

Influenza activity in mainland China: Chinese national sentinel surveillance for influenza virus comprises more than 554 sentinel clinics in China and 408 collaborating laboratories for identifying the respiratory viruses. We retrieved the weekly number of outpatient per 100 visits for ILI to sentinel clinics and the positivity rate of influenza virus was obtained from the Chinese National Influenza Center [9]. Then we calculated weekly ILI+ proxy using these data streams for mainland China. We also had an opportunity to classify these data to explore spatially for Southern mainland China and Northern mainland China as defined via latitudes by the Chinese National Influenza Center [9].

Influenza activity in Hong Kong: We collected the weekly proportion of outpatient consultations due to influenza-like illness (ILI) and the weekly proportion of sentinel specimens tested positive for influenza viruses in Hong Kong. The Centre for Health Protection tracks a sentinel network of private doctors and reports weekly rates of outpatient consultations for ILI, per 1000 consultations [10]. The Public Health Laboratory Services branch reports the weekly proportion of respiratory specimens testing positive for influenza by type/subtype [11]. We calculated weekly ILI+ proxy using both the data streams for Hong Kong.

Influenza activity in Taiwan: Taiwan national sentinel surveillance for influenza virus comprises more than 275 sentinel clinics and 12 collaborating laboratories for identifying the respiratory viruses [12]. We retrieved the positivity rate of influenza virus was obtained from the Taiwan Influenza Express Achieve [13]. Furthermore, we collected the weekly number of outpatient visits for ILI as well as weekly proportion of outpatient visits for ILI to sentinel clinics. Then we calculated weekly ILI+

proxy using these available data streams for Taiwan.

Influenza activity in South Korea: We obtained the weekly number of patient for ILI per 1000 visit from the 200 sentinel primary care clinics [7]. We also collected the influenza virus detection rate which was determined by reverse transcription polymerase chain reaction in clinical samples. We retrieve these data from the Weekly Sentinel Surveillance Report of Korea Disease Control and Prevention Agency [14]. Finally, we calculated weekly ILI+ proxy using these available data streams for South Korea.

Influenza activity in Singapore: We obtained Singapore influenza syndromic and virological data from FluID [15] and FluNet [16] to calculate the indicator to represent the influenza activity of Singapore. Because the weekly numbers of total clinical visits were not available, we could not get ILI+ rate. We multiplied the weekly numbers of clinical visits with influenza-like illness (ILI) symptoms by the proportion of positive laboratory test for influenza virus to calculate the indicator for weekly Influenza activity for Singapore.

Influenza activity in Japan: We retrieved the number of reported cases per sentinel clinic and influenza virus positive rate from the Weekly Data Achieve of Japanese National Institute of Infectious Disease [17]**.** We obtained the number of cases of seasonal influenza which is diagnosed by the physician based on clinical symptoms or laboratory findings from 5000 sentinel medical institutions in Japan [18]. The total number of weekly consultations per sentinel clinic was not provided, therefore we approximated the ILI + Proxy for Japan as the product of the laboratory positive rate and number of cases per sentinel clinic.

Influenza activity in Italy: We obtained influenza epidemiology surveillance data and laboratory surveillance data from FluID [15] and FluNet [16] to compute the proxy representing the influenza activity of Italy. Because the weekly numbers of total clinical visits were unavailable, following

Kramer and Shaman [19], we obtained the clinical visits with influenza-like illness (ILI) symptoms and the proportion of positive laboratory tests for influenza virus to calculate weekly case proxy for Italy.

Influenza activity in Germany: Similar to Italy, we obtained influenza syndromic and virologic data from FluID [15] and FluNet [16] to calculate the case proxy to represent the influenza activity in Germany. Due to the unavailability of weekly ILI cases, we used weekly acute respiratory infection (ARI) cases to calculate weekly flu case proxy for Germany.

Influenza activity in the UK: We obtained the virological and syndromic data on influenza from the weekly national flu reports of primary care and microbiological surveillance [20] published by Public Health England (PHE). While weekly GP Influenza-like-illness consultation rates are reported by around 360 GP practices across England and weekly influenza specimen tested positive rates were reported through Respiratory DataMart System [21]. We used these data streams to calculate the ILI+ proxy for the UK (particularly for England).

Influenza activity in the USA: We retrieved the weekly number of outpatient visit per 100 visits for ILI to sentinel clinics (per sentinel site) and the positivity rate of influenza virus was obtained from the US centers for disease control and prevention [22] and we calculated weekly ILI+ proxy for the USA.

Influenza activity in Australia: We obtained influenza epidemiology surveillance data and laboratory surveillance data from FluID [15] and FluNet [16] to compute the ILI+ proxy representing the influenza activity of Australia.

2. Collection of location- or country-wise data on PHSMs for COVID-19

We retrieved the temporal information on public health and social measures (PHSMs), implemented over the course of the pandemic for these countries in response to COVID-19 pandemic. We collected the implementation timing and duration for each measure, and classified them into case-based, community-wide and travel-realted control measures. The case-based interventions refer to the measures at individual level including case identification and isolation, and quarantine of contacts of confirmed cases. The community-wide measures target the population as a whole including all physical measures of social distancing and behavioural changes. The travel-realted measures include travel restrictions, port control and inbound traveller screening [23]. The summary of these interventions with their timing and duration for each country are provided in Table S1 and available online [24].

3. Collection of data on sessional routine vaccination (timing, duration and coverage) and yearly number of visitors for the studied countries/ locations.

We retrieved the information on timing, duration and coverage of seasonal influenza vaccination program (routing annual vaccination program) from the available government/media reports and literatures in the studied countries/ locations during 2017-2022 (Table S2). For the countries/ locations with missing or unavailable (e.g., Mainland China and South Korea) information on the timing and duration, we considered the routine vaccination period as October – December. While some of the countries/ location had information on the start of such vaccination program, but the end timings were not clearly identified from the reports or literatures therefore, we restrict the duration for maximum of 3 months (~13 weeks) for such countries/ locations for each season. We calculated the seasonal routine vaccination rate from the duration and vaccination coverage and assumed to be same throughout the study period for each country/location if no information of enhanced seasonal vaccination reported during this period (e.g., Italy reported an enhanced sessional influenza vaccination during 2020-21 seasons).

We also collected the information on yearly number of visits in the studied countries/ locations during 2017-2022 from the reports or websites, available for respective governments and travel agencies (Table S3). We considered the travel in and travel out of any country/location in a year approximately same and hence the respected seeding of infectious individuals (i.e., weekly number of infectious travelers since the COVID-19 PHSMs assumed to be relaxed, ϵ_0) (Appendix Section 6.1).

4. Log-linear regression model for transmissibility, R_t

We first used the branching process model under Bayesian inferential framework to estimate the real-time effective reproduction number at time t, denoted by R_t, represents an instantaneous measure of transmissibility and defined as average number of secondary infections generated by a typical primary infectious case at time t. When R_t exceeds 1, the epidemic will continue to spread. We estimated R_t for each location/ country from daily ILI+ proxies, derived by using a spline interpolation on the observed weekly ILI+ proxies [5,25]. Here we considered the serial interval distribution as the Gamma distribution with mean 3.2 days and standard deviation 1.3 days [26]. Where, R_0 is the basic reproduction number (a measure of initial transmissibility), S_t is the proportion of susceptible individuals at time t and I_t is the number of infectious cases at time t. In theory, R_t depends on the initial transmissibility (i.e.; R_0) and the susceptibility at that time t (i.e., S_t), i.e., $R_t = R_0 S_t$. Hence, $R_t = R_0$ when all individuals are susceptible ($S_t = 1$). R_t is also subject to be decreased over time along the depletion of susceptibility (S_t). In reality, R_t may be further modified by other factors, such as introduction of control measures [27-31]. Following this hypothesis, we then proposed a log-linear multivariable frame work to quantify the impact of the COVID-19 PHSMs on transmissibility of influenza virus in these locations.

Consider, C_t is a variable indicating the timing (start and duration) and stringency (measure of rigorousness) of the PHSMs implemented against COVID-19. Where we explore some forms of C_t by accounting the temporal stringency of PHSMs across the pandemic. The simplest form of C_t,

we considered as a categorical variable,

$$C_t = \begin{cases} 1, & day\ t\ is\ under\ COVID-19\ NPIs\ impemented\ period \\ 0, & day\ t\ is\ before\ COVID-19\ NPIs\ impemented\ period \end{cases}.$$

But this form doesn't consider the temporal changes in the stringency of PHSMs which implemented across the pandemic. Hence, we formulated C_t by accounting the stringency of interventions that were in force on time t (day/week). We first summarized in a number of PHSMs from all case-based, community-wide and travel-realted interventions (as listed in Table S1), implemented at the country or territory level in response of COVID-19 control over the time for these locations/ countries (except mainland China), then used C_t as the number of interventions that were in force at time t. For mainland China, we formulated C_t using the available city-level interventions data for 260 Chinese cities [32] as the sum of the proportions of cities that implemented a certain type of PHSM on time t. The PHSMs for mainland China include 7 types of interventions: isolation of confirmed cases, insolation of suspected cases, suspension of travel between cities (i.e., inter-city travel ban), suspension of intra-city public transport (intra-city travel ban), closure of public services (e.g., shopping malls, restaurants), closure of entertainment and public gathering venues (e.g., bar, cinema, park), and recruitment of governmental staff and volunteers to enforce quarantine (i.e., social mobilization) [32].

Therefore, C_t can be expressed in general as

$$C_t = \begin{cases} \sum_{k=1}^{7} \eta_k(t)/N, & mainland\ China \\ \sum_{l=1}^{3} \psi_l(t), & otherwise \end{cases}$$

Where, $\eta_k(t)$ is the number of cities in mainland China where the k-th intervention ($k = 1, 2, \ldots, 7$) was in force on day t, and N is the total number of cities (260). $\psi_l(t)$ is the number of PHSMs that were in force on day t of l-th intervention type (case-based, community-wide, and travel-based, i.e., $l = 1, 2, 3$) for the regions/countries other than mainland China.

Therefore R_t can be expressed as: $R_t = R_0 S_t e^{\lambda C_t}$; where, λ is a coefficient indicating the effect of PHSMs, a significant negative λ ensures the reduction in R_t by the PHSMs. As susceptible proportion, $S_t = S_0 - h_t = S_0(1 + \alpha h_t)$; where, S_0 is initial proportion of susceptibles in the population, $\alpha = -1/S_0$ is a constant, $h_t = \sum_{x=1}^{t-1} I_x$ is a proxy variable indicating the depletion of susceptibles at time t. Using Taylor series expansion, we have $S_t \approx S_0 e^{\alpha h_t}$ and we get, $R_t = R_0 S_0 e^{\alpha h_t} e^{\lambda C_t}$. Further, taking logarithm we have the log-linear multivariable regression model as $ln(R_t) = K + \alpha h_t + \lambda C_t$; where, $K = ln(R_0 S_0)$. $\alpha(< 0)$ and λ are regression coefficients for the depletion of susceptible and PHSMs, respectively. Finally, the maximum overall changes in transmissibility were evaluated by the metric $\sum_{t=1}^{n}(1 - e^{\lambda C_t})/n \times 100\,\%$, through exploring different lengths (n) of R_t time series.

5. Susceptible-Exposed-Infected-Recovered (SEIR) transmission model and simulation of the attack rate

We formulated a standard Susceptible-Exposed-Infected-Recovered (SEIR) transmission model to simulate the influenza activity (attack rate) under different counterfactual scenarios. Consider a standard SEIR model $dS/dt = -\beta_t SI$, $dE/dt = \beta_t SI - \sigma E$, $dI/dt = \sigma E - \gamma I$, $dR/dt = \gamma I$ where S, E, I, R denote the proportion of susceptible, exposed, infectious, and recovered individuals respectively. β_t is the transmission rate. The rate of becoming infectious is σ and the recovery rate γ. We set the value of σ and γ to the ranges of (0.50, 0.63) and (0.50, 0.70) per day respectively (Table S5), which ensures the mean generation time in (3.0, 4.0) days for influenza virus [33-35] which allowed to have the best mimic of the influenza activity (Figure S3). We have chosen the initial condition such that there is around same observed attack rate (by best fitting to cumulative number of cases) before the start of PHSMs implementation. Further, if the PHSMs were implemented after peak, we allowed a constrain of same interval between peak and the start of the PHSMs additionally. Then the simulation was performed to reproduce the influenza activity, which is assumed to be proportional to the compartment I [1].

For exploring the counterfactual scenarios, we reconstructed the transmission rate (β_t) from effective reproduction number (R_t), as latter metric is highly influenced by depletion of susceptible. Using the above SEIR model assumption, we have $R_t = \beta_t/\gamma \times S_t$. Further, we know $R_t = R_0 S_t e^{\lambda C_t}$ (as derived in Appendix section 2). Therefore, β_t can be expressed as $\beta_t = \beta_0 \, e^{\lambda C_t}$, since, $\beta_0 = R_0\gamma$ when $S_0 \approx 1$. Then using the estimated regression coefficients (K, α and λ) in section 2, we have $R_0 = e^K/S_0$. Then we evaluated the reduction in the attack rate (total infections) by comparing the simulated epi-curves generated with estimated λ vs $\lambda = 0$ (indicating no impact of PHSMs) in $\beta_t = \beta_0 \, e^{\lambda C_t}$. We further considered a possible range of initial susceptibility S_0 a measure of pre-immunity in the population and conduct a sensitivity analysis. We allowed 0% to 30% of immunity at the start of the epidemic and performed the simulation under each scenario. We presented the results in the main text based on no pre-immunity and for other realization of S_0 presented in Table S5.

We extended this modeling framework to address the sustained zero circulation of influenza viruses globally during COVID-19 pandemic. In fact, the 2020 summer influenza season is missed in the tropical regions, where influenza circulates year-round and then the successive seasons were missed around the globe. Here, we considered Hong Kong for example. We used the same SEIR model as described above to first mimic the two peaks (winter and summer) allowing the immunity wining and infection seedings. Then explore the impact of COVID-19 PHSMs, we allowed a plausible range of estimates of λ from -0.0 to -0.3 (containing maximum values as estimated in the regression model for different locations/ countries). We also considered the reasonable infections seeding (ι), allowing up to 200 infections per day per million. Finally, we quantified the minimum reduction in λ defined as $\lambda_{min} = min\,(|\lambda|)$, which had the potential to contain the zero-influenza circulation (Figure S4).

6. Predictive mechanistic model to infer next influenza seasons

In practice, maintaining λ_{min} for a longer time will be difficult to sustain, and as immunity to COVID-19 gradually increases through vaccinations and infections, it is likely that PHSMs will progressively be relaxed. To infer the upcoming influenza seasons when COVID-19 PHSMs are relaxed, we developed a stochastic susceptible-vaccinated-infectious-recovered-susceptible (SVIRS) model-based inferential framework to fit these influenza activity proxy data (e.g., ILI+ proxy) first and conducted forecasting afterwards.

6.1 Model construction and specification

A SVIRS model divides the population (N) into those individuals susceptible to infection (S), vaccinated (V), infectious (I) and recovered (R). The transitions of individuals between compartments were specified by different rates. We formulate the following stochastic SVIRS model with immunity and demography, which is illustrated in Figure S1,

$$S(t+\delta t) = S(t) + \Delta B - \Delta H_S - \Delta V_S + \Delta W_V + \Delta W_R - \Delta D_S$$

$$V(t+\delta t) = V(t) + \Delta V_S - \Delta H_V - \Delta W_V - \Delta D_V$$

$$I(t+\delta t) = I(t) + \Delta I_{in} - \Delta I_{out} + \Delta H_S + \Delta H_V - \Delta R_I - \Delta D_I$$

$$R(t+\delta t) = R(t) + \Delta R_I - \Delta W_R - \Delta D_R$$

Euler approximation is used to provide the solution of the model with the step size, δt, fixed to be 1 day. The above formulas show the process increments noted by Δ at time t. Herein ΔB is the number of births, ΔV_S is the number of people vaccinated, ΔH_S and ΔH_V are the incidence number from the susceptible and the vaccinated compartment respectively, ΔR_I is the number of recovered individuals, ΔW_V and ΔW_R are the number of people with vaccination-acquired immunity and infection-acquired immunity waned respectively, ΔI_{in} and ΔI_{out} are the numbers of infected individuals (visitors) imported and exported respectively, and ΔD_i is the number of natural deaths that occur in the population of compartment i. These processes of increment follow the specific probabilistic distributions as shown below,

$$\Delta B \quad \sim Poisson\big(\delta t \cdot \theta \cdot N(t)\big)$$

$$(\Delta H_S, \Delta V_S, \Delta D_S) \quad \sim Multinomial(S(t); \xi(t), \tau(t), \mu)$$

$$(\Delta H_V, \Delta W_V, \Delta D_V) \quad \sim Multinomial(V(t); (1-\omega)\xi(t), \rho_V, \mu)$$

$$(\Delta R_I, \Delta D_I) \quad \sim Multinomial(I(t); \gamma, \mu)$$

$$(\Delta W_R, \Delta D_R) \quad \sim Multinomial(R(t); \rho_R, \mu)$$

$$\Delta I_{in} \quad \sim Poisson\big(\delta t \cdot \epsilon(t)\big)$$

$$\Delta I_{out} \quad \sim Poisson\big(\delta t \cdot \epsilon(t)\big)$$

The $Multinomial(X; r_1, \dots, r_k)$ denotes that the numbers of individuals transiting with rates $r_1, \dots, r_k$, where they together with those remaining in the compartment follow a multinomial distribution with X individuals with probability $\left(p_1, \dots, p_k, 1 - \sum_{i=1}^{k} p_i\right)$ where $p_i = \delta t \cdot r_i$. The birth rate (θ) and death rate (μ) are fixed as 1/80 year^{-1}. We assumed the infectious duration of influenza ($1/\gamma$) to be 4 days, and the infection-acquired immunity duration ($1/\rho_R$) to be 52.14 weeks. The details of the model parameters and variables are illustrated with their pre-defined values and mode of estimation in the Table S4.

Along with the routine vaccination programme implemented in different countries/locations, we considered a one-off vaccination programme, needed to fight against the in response to the surged influenza epidemic in the upcoming seasons when the COVID-19 PHSMs are relaxed. $\tau(t)$ is the vaccination rate per week of the total susceptible population over time and is defined as follows,

$$\tau(t) = \tau_r \mathbb{I}\{\tau_r^S < t < \tau_r^E\} + \tau_o \mathbb{I}\{\tau_o^S < t < \tau_o^E\},$$

where τ_r denoted the vaccination rate constant under the routine vaccination programme, and $\mathbb{I}(\tau_r^S \le t \le \tau_r^E)$ is the indicator function for the routine vaccination which starts at time τ_r^S and ends at time τ_r^E. The vaccination rate was evaluated from the information on timing and coverage for routine vaccination for each country/location as provided in Table S2. The vaccination rate constant for the one-off vaccination programme (τ_o), and the corresponding starting time (τ_o^S) and ending time (τ_o^E), would be specified when assessing the effect of one-off vaccination strategy in the forecast simulation (i.e. τ_o is kept 0 for the period in model fitting). Furthermore, the vaccination-acquired immunity was shown to last for > 6 months [36], and it was assumed to be 9

months (i.e. $1/\rho_V$ = 47 weeks) in this model.

We specify the force of infection ($\xi(t)$), the rate of a typical susceptible acquiring influenza infection [37], as follows,

$$\xi(t) = \beta_e(t)\frac{I(t)}{N(t)}$$

Herein $\beta_e(t)$ is the transmission rate of influenza virus. Under the "leaky" assumption, force of infection for vaccinated individuals would be $(1-\omega)\xi(t)$ instead, where ω is the vaccine efficacy assumed to be 0.59 [38]. Based on the next-generation matrix, such SVIRS model would give the following time-varying reproduction number, $R_e(t) = \beta_e(t)/(\gamma+\mu)$, defined as the expected number of secondary cases transmitted by an index case in an entirely susceptible population at time t [39]. For the ease of interpretation, we modelled $R_e(t)$ instead of $\beta_e(t)$. Since the influenza epidemics are seasonally forced, we introduced sinusoidal functions with a period of 52.14 weeks for the seasonal variation in $R(t)$ as follows,

$$R_e(t) = R_{e,min} + \left(R_{e,max} - R_{e,min}\right) expit\left(\alpha_0 + \alpha_1 \sin\left(2\pi\frac{t}{52.14}\right) + \alpha_2 \cos\left(2\pi\frac{t}{52.14}\right)\right)$$

The parameters α_0, α_1 and α_2 are the coefficients for the seasonality of $R_e(t)$, where $expit(\cdot)$ is the logistic sigmoid function (i.e., the inverse of logit function). $R_{e,min}$ and $R_{e,max}$ provided the range of $R_e(t)$, which are the possible minimum and maximum time-varying reproduction number for influenza infection respective. $R_{e,max}$ is fixed as 3 and $R_{e,min}$ is fixed as 1.

The surge in influenza epidemics after COVID-19 PHSMs relaxation worldwide may lead to the increase in the cross-border population mobility, leading to the importation risk (infectious travelers), which can be the source of outbreak locally. We defined the weekly number of infectious travelers ($\epsilon(t)$) as 0 for the period during model fitting (pre-pandemic) and until before COVID-19 PHSMs were relaxed, and as ϵ_0 since the COVID-19 PHSMs assumed to be relaxed. We assumed the country-specified ϵ_0 to be 0.1% (0.02% and 0.5% as sensitivity analysis) of the visitor arrivals

based on the annual visitor arrival data (Table S3). Besides, the increment ΔI_{out} would be 0 when the number of infectious individuals in compartment $I < \epsilon(t)$, ensuring the baseline seeding in population given imported infectious travelers.

6.2 Likelihood and Parameter estimations

We adopted the partially observed Markov process (POMP) model to conduct likelihood evaluation and parameter estimations for the SVIRS model (Table S4 and S6). In general, a POMP model would have the following joint probability density function [40],

$$f_{X_{0:N},Y_{1:N}}(x_{0:N}, y_{1:N}; \Theta) = f_{X_0}(x_0; \Theta) \prod_{n=1}^{N} f_{X_n|X_{n-1}}(x_n|x_{n-1}; \Theta) f_{Y_n|X_n}(y_n|x_n; \Theta)$$

Herein $X_{0:N} = X_0, \dots, X_N$ are the hidden states involving the Markov process at times $t_0, \dots, t_N$, and Θ is the parameter vector. The hidden states $X_{0:N}$ is only observed by way of another process, $Y_{1:N} = Y_1, \dots, Y_N$, which is assumed to depend only on the hidden state at the corresponding time. The POMP model would have a joint probability density function involving the probability density of the initial states, f_{X_0}, the transition probability density of the hidden states given the Markov property, $f_{X_n|X_{n-1}}$, and the probability density of measurement, $f_{Y_n|X_n}$. Since only the process $Y_{1:N}$ is observed, the marginal likelihood ($\mathcal{L}(\Theta)$) at the data $y^*_{1:N}$ would be,

$$\mathcal{L}(\Theta) = f_{Y_{1:N}}(y^*_{1:N}; \varphi) = \int f_{X_{0:N},Y_{1:N}}(x_{0:N}, y^*_{1:N}; \Theta) dx_{0:N}$$

In our model, the hidden states are the compartments under the SVIRS model, where the transition probability densities between compartments were already specified previously. Regarding the measurement, as the influenza activity is always only partially detected limited to the surveillance capacity, the observed incidence number ($H^{obs}(t)$) is assumed to follow negative binomial distribution conditional on the incidence number ($H(t)$, i.e. cumulative sum of ΔH_V and ΔH_V at week t) with a mean of $\phi H(t)$ and a variance $\phi H(t)\{1 + \sigma\phi H(t)\}$, where ϕ and σ are the scaling factor and overdispersion parameter, respectively.

Such likelihood can be evaluated by sequential Monte Carlo, as known as particle filtering, which is a plug-and-play method that only requires simulations of particle [41]. Iterated filtering (IF2), which is a technique for maximizing likelihood obtained by filtering in software package "POMP", is used for parameter estimation [40]. The algorithm incorporates perturbation of model parameters during iterations, and the intensity of which would gradually reduce as the iterations proceed. As a theoretical result, the swarm of particles would concentrate near the maximum likelihood estimates. We used 1000 particles with 200 iterations for the IF2 algorithm for likelihood optimization. The "*cooling.fraction.50*", which is a hyperparameter for the IF2 algorithm indicating the relative magnitude of the perturbations after 50 iterations, is set as $\sqrt{0.5}$ to have the magnitude of perturbation reduced by half for every 100 iterations, allowing exploration in the parameter space. We also facilitated the search of estimates with global maximum likelihood by initializing the IF2 algorithm with 1000 sets of random combinations of parameter values using the Latin hypercube sampling technique.

However, IF2 does not evaluate the true likelihood during optimization, but instead the likelihood subject to random perturbations to the parameters, hence the algorithm may not always return the maximum likelihood estimates [40,42,43]. In light of this, we evaluated the likelihood of the parameter space near the estimates by particle filtering to obtain the likelihood profile, and applied the Monte Carlo-adjusted profile (MCAP) algorithm to the likelihood surface [44]. Despite that the particle filtering is a Monte Carlo algorithm which gives noisy estimates of likelihood, the MCAP algorithm can provide likelihood-based parameter estimates and construct confidence interval while taking Monte Carlo error into account. Such approach allows us to verify the IF2 estimates and obtain the corresponding 95% confidence intervals. We would replace the IF2 parameter estimates with MCAP estimates as our parameter estimates if the MCAP estimates give a higher likelihood. The estimates would also be assessed by simulating the fitted model.

On the other hand, since our model is an ODE system, its solution depends on the initial values of

compartments, X_0. To reduce the number of parameters to be estimated, the initial value of S, V, I, R were arbitrarily fixed to be 0.699, 0.001, 0.001 and 0.299. After that, the model would have simulation for 10 years ahead as warm-up before model fitting to alleviate the effect of mis-specification of initial values to the solution.

6.3 Data rescaling

Since the measurement of the SVIRS model is weekly incidence in terms of number of cases, we approximate $H^{obs}(t)$ by multiplying the weekly ILI proxy with a rescaling factor, κ, which could lead to better optimization performance of such models [19]. The rescaling factor can be different by location/ country and is kept constant over seasons, where the value of which is determined by first calculating the range of scaling values yielding a total attack rate of 20% in season 2017/18 and 2018/2019, and choosing the minimum of the scaling values.

6.4 Model fitting for Northern and Southern Mainland China

The model fitting in general was based on the ILI proxy without influenza virus subtyping by country/location. However, we found the prolonged influenza activity or bi-peak epidemic in season 2018/19 in Northern and Southern Mainland China, as well as South Korea. Thus, we fitted the model to the data based on ILI proxy by influenza A and B subtype separately assuming the transmission dynamics of influenza A and B viruses were independent, and combined the result in our preliminary analysis. We found that this approach provided a better model fit to data of Northern and Southern Mainland China but not to the data of South Korea. Hence, the results of Northern and Southern Mainland China presented were based on the combination of result by influenza virus subtype.

6.5 Predictive simulation for upcoming flu seasons

After parameter estimation, we used the fitted model to simulate the future seasons in these locations under the intervention of COVID-19 PHSMs. Given the influenza activity was undetectable since

February 2020, we assumed that the intervention would reduce the time-varying reproduction number of influenza for a certain percentage since February 2020, which would recover when the PHSMs are relaxed. Considering $R_{e,0}$ as the initial reproduction number typically measured for influenza season during pre-COVID-19 pandemic where $R_{e,0} = max(R_e(t))$, we set the minimum proportion of reduction in $R_e(t)$ as $p^{reduced} = 1 - 1/R_{e,0}$, so that the reduced time-varying reproduction number, ${R_e}^{reduced}(t) = (1 - p^{reduced})R_e(t)$ smaller than 1, and hence the disease would stop spreading in the population during the period with PHSMs implementation.

When the PHSMs last for a longer period, the population would have the waning protection against influenza and become more susceptible, hence a larger influenza outbreak when the PHSMs are relaxed. Besides, the $R_e(t)$ would only partially recover as the PHSMs would not be fully relaxed given the raising public awareness of hygiene and the latest situation of the emerging COVID-19 virus with its successive variants. Thus, the magnitude of the future influenza seasons primarily depends on the effective duration (v) of COVID-19 PHSMs as well as the proportion of recovered in $R_e(t)$ (denoted as $p^{recovered}$), where the recovered time-varying reproduction number due to relaxation of PHSMs is defined as ${R_e}^{recovered}(t) = {R_e}^{reduced}(t) + p^{recovered}\left(R_e(t) - {R_e}^{reduced}(t)\right)$ at time t. $p^{recovered}$ close to 0 represents that the $R_e(t)$ is just slightly recovered (i.e., ${R_e}^{recovered}(t) \approx {R_e}^{reduced}(t)$), while $p^{recovered}$ close to 1 represents that the $R_e(t)$ is almost recovered (i.e., ${R_e}^{recovered}(t) \approx R_e(t)$). We predicted the influenza outbreak by simulating the situations when the PHSMs will be relaxed in October 2021, April 2022, and October 2022 to assess the effect of COVID-19 PHSMs on the influenza summer peak and winter peak specifically, which corresponds to v of 21, 27, 33 months respectively, and when $p^{recovered} \in \{0.1, 0.2, \ldots, 1.0\}$.

Our model also allows us to suggest a possible proactive influenza vaccination programme against the next influenza season which can potentially mitigate the risk of large outbreaks. Considering the

different levels of pre-immunity in the population during 2017-2019 seasons, we defined a threshold of the mean trajectory for the observed incidence rate to indicate the epidemic onsets. We set these thresholds at the 75th quantile of incidence rate for mainland China, Hong Kong, South Korea, Japan, Italy, Germany, United States and United Kingdom; and the 50th quantile for Taiwan and Singapore. To assess the effect of one-off vaccination programme, we determined τ_o^E as the time after COVID-19 PHSMs period when the predicted season started developing given the threshold. Thus, we conducted simulation for the predicted upcoming season observed within 1 year when the vaccination rate $\tau_o \in \{0.01, 0.02, \dots, 0.1\}$, and the vaccination period, $\tau_o^L = \tau_o^S - \tau_o^E$, varies from 0 week (no vaccination) to 26 weeks (6 months), while $p^{recovered}$ is assumed to be 1.0 when assessing the effect of vaccination programme.

6.6 Assessment of excess burden for upcoming seasons

We estimated the excess infection burden for upcoming seasons by defining two quantities, *δ-fold rise* in '*peak magnitude*' and '*epidemic size*'. Where the *peak magnitude* indicates the maximum of trajectory of the simulated incidence rate ($H(t)$) and *epidemic size* indicates cumulative incidence of the predefined epidemic, respectively. We considered these measures, *peak magnitude* and *epidemic size* for 2017-19 seasons as the baseline scenarios of pre-COVID-19, and compare with the respective measures derived for upcoming seasons within 1 year after the relaxation of COVID-19 PHSMs. To quantify the excess burden, the *δ-fold rise* in *peak magnitude* is defined as the ratio of the baseline *peak magnitude* to the *peak magnitude* of upcoming season. Similarly, the *δ-fold rise* in *epidemic size* is defined as the ratio of the baseline *epidemic size* to the *epidemic size* of upcoming season. Therefore, $\delta = 1$ indicates the no excess burden and $\delta > 1$ indicates the increased burden of δ-fold.

7. Figures S1 to S13

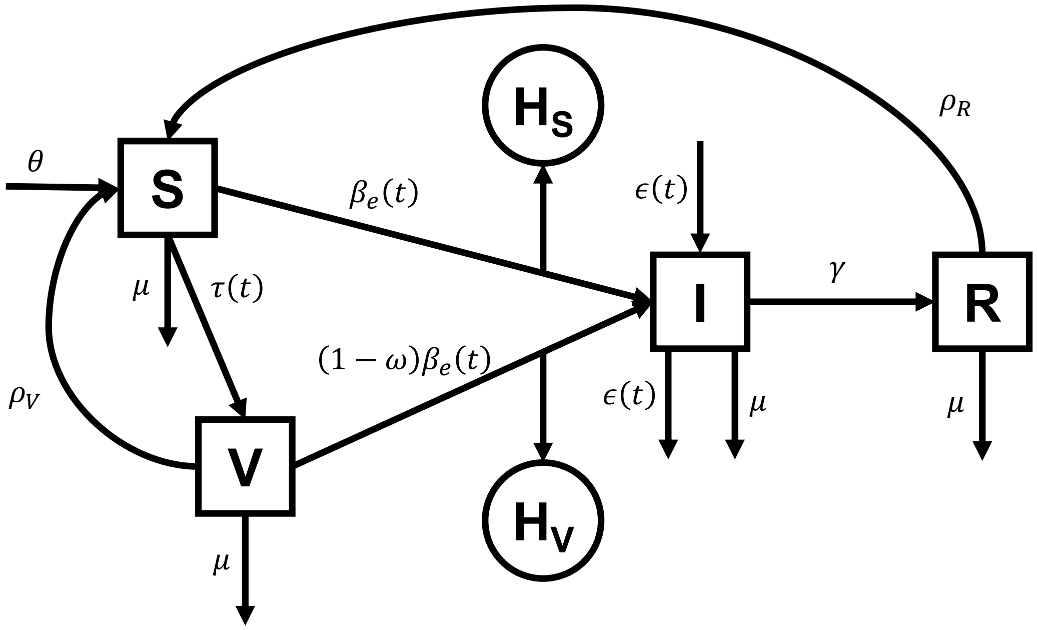

Figure S1: Schematic illustration of the mechanistic model. The population is divided into 4 compartments, where S, V, I and R represent the susceptible, vaccinated, infectious and recovered individuals in the population respectively. θ and μ were the birth rate and death rate respectively. $\tau(t)$ denotes the vaccination rate, whereas the vaccine-acquired immunity would wane at a mean of $1/\rho_V$ weeks. The process increment H_S is the incidence number from compartment S to I with the transmissibility $\beta_e(t)$, and the process increment H_V is the incidence number from compartment V to I with the transmissibility $(1-\omega)\beta_e(t)$ under the "leaky" vaccine assumption of vaccine efficacy ω. The infectious population had the infectious period of a mean of $1/\gamma$ weeks and would transit to compartment R when recovered, while their infection-acquired immunity would wane at a mean of $1/\rho_R$ weeks later and they became susceptible again. Besides, $\epsilon(t)$ is the seeding of infectious individuals considering the surge in infectious travelers when the COVID-19 PHSMs were relaxed.

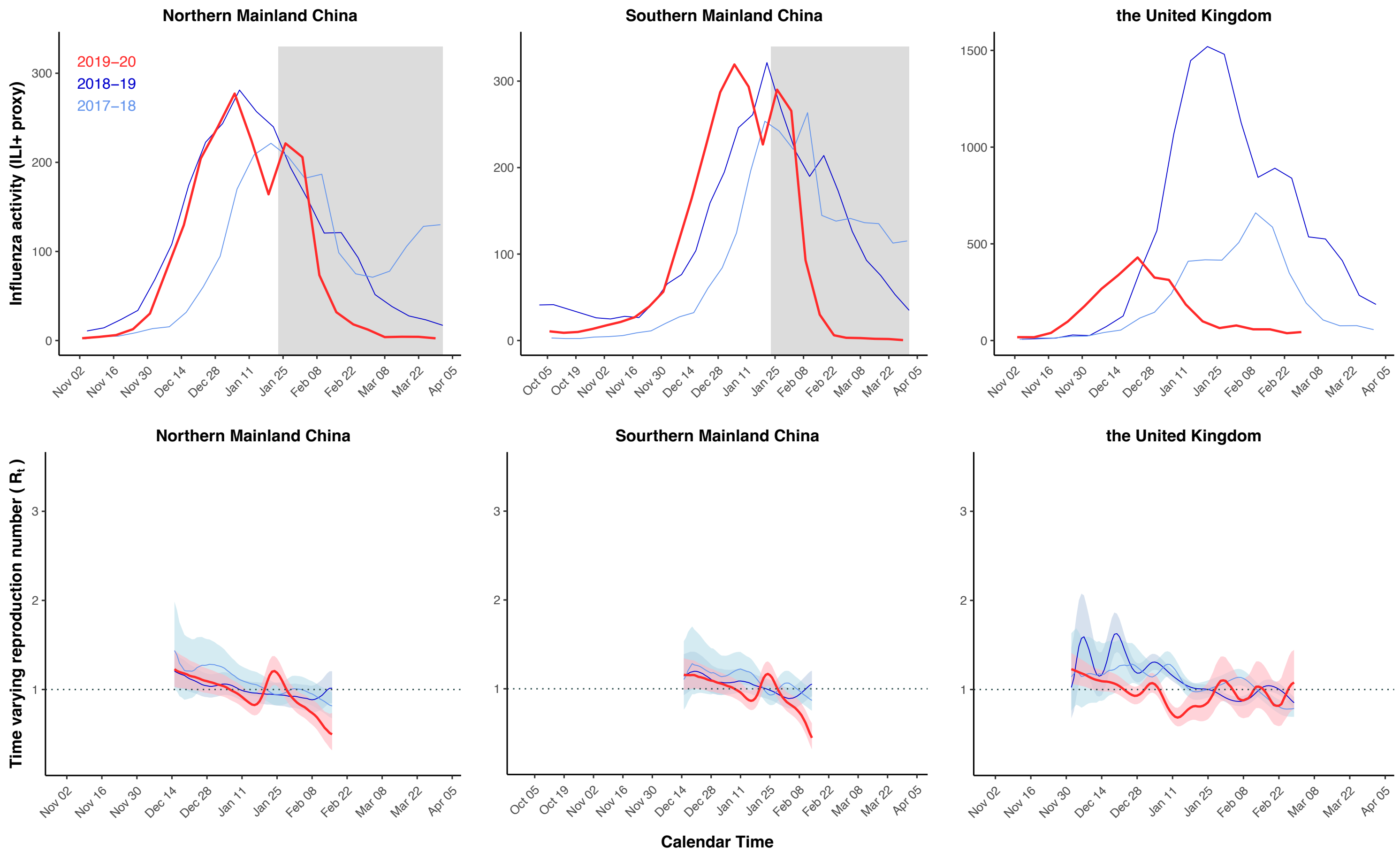

Figure S2. **Reduction in influenza activity (weekly ILI+ Proxy) and transmissibility (R_t) for the Northern and Southern mainland China, and the United Kingdom. during 2019-20 season by public health social measures (PHSMs) in response to COVID-19 pandemic in 2020.** The time series of ILI + Proxy are for the 2019-20 season (in red), 2017-18 season (in light blue) and 2018-19 season (in blue) in these locations. The grey shaded regions are the indicator of timing of COVID-19 pandemic for each location, identified for the effective impact of COVID-19 PHSMs on influenza as the indicator of pre- and during pandemic scenarios. Note: The influenza activity in the United Kingdom started declining from late December 2019 whereas the countrywide COVID-19 PHSMs were implemented much later

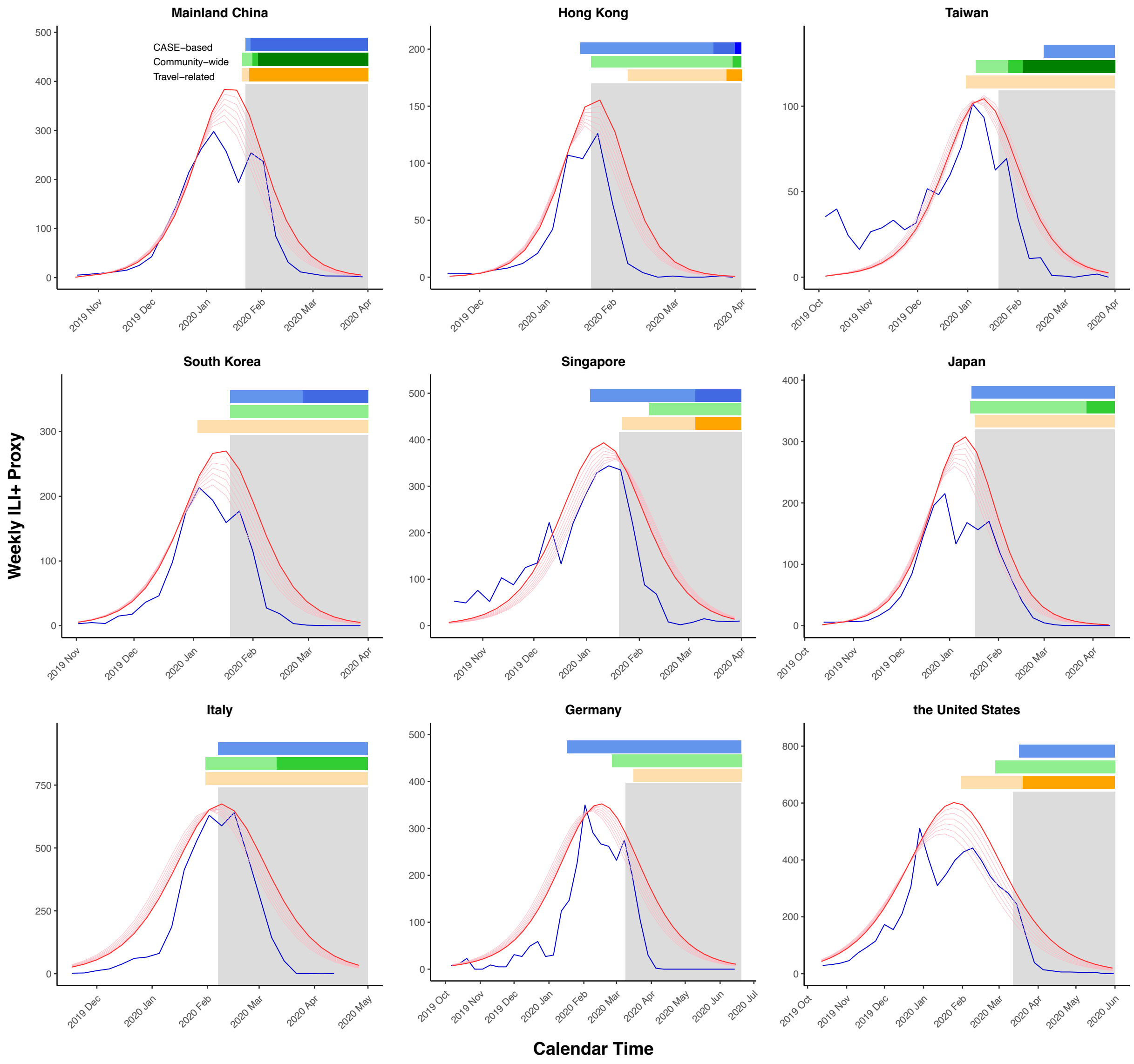

Figure S3 Counterfactual simulation of influenza attack rates in different locations (9 regions/ countries) across the globe during 2019-20 season considering no impact of the public health social measures (PHSMs) in response to COVID-19 pandemic in 2020. The blue lines indicate the number of observed cases (weekly ILI+ proxy). The simulated incidence of influenza viruses without implementation of COVID-19 PHSMs are in red curves with the best fit (in solid red curves) on the observed data (till the exponential phase). The timing and the magnitude indicators of PHSMs are classified in case-based (in blue shades), community-wide (in green shades) and travel-related (in orange shades). The grey shaded regions are the indicator of timing of COVID-19 pandemic for each location, identified for the effective impact of COVID-19 PHSMs on influenza as the indicator of pre- and during pandemic scenarios.

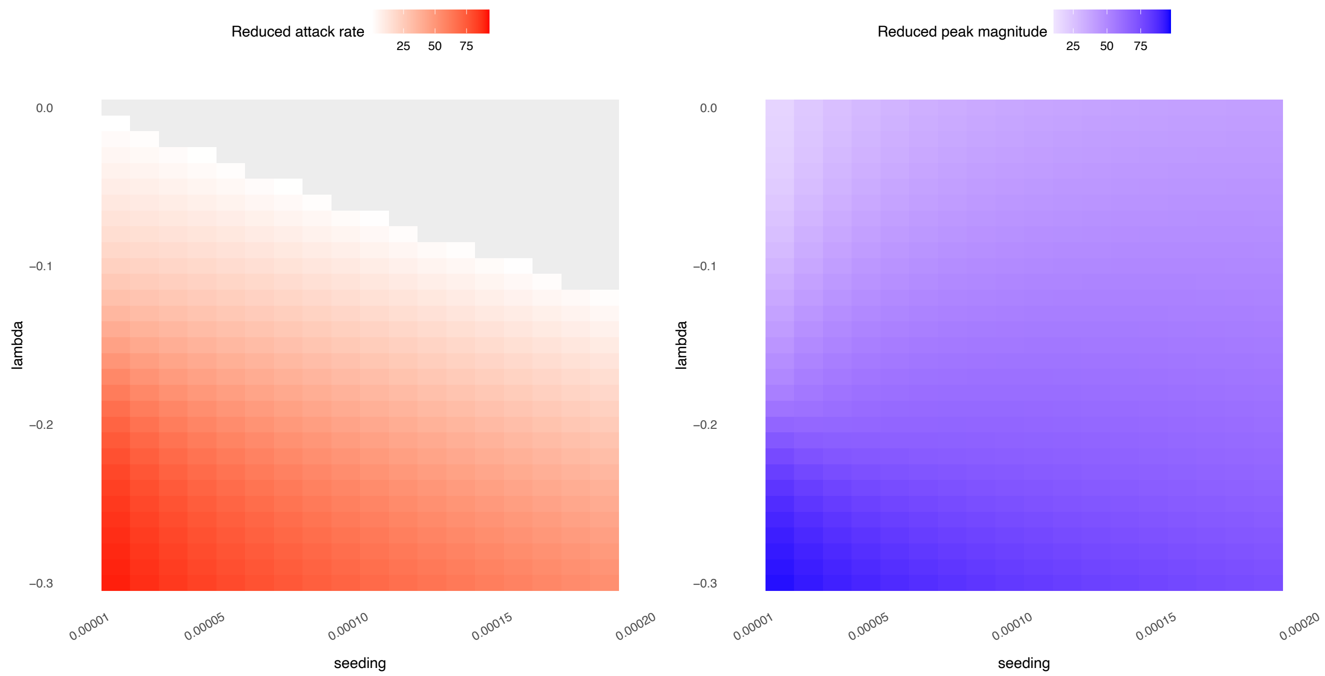

Figure S4: **Reduction in attack rate (infections) and the peak magnitudes under the impact of the public health social measures (PHSMs) and possible seeding for Hong Kong (as an example).** The impact of the PHSMs is measured here as the λ values, indicating under seeding around 1 infection per day per million could reduce to the maximum and maintained the zero sustained infection when $\lambda \approx -0.3$, and could be higher with higher seeding.

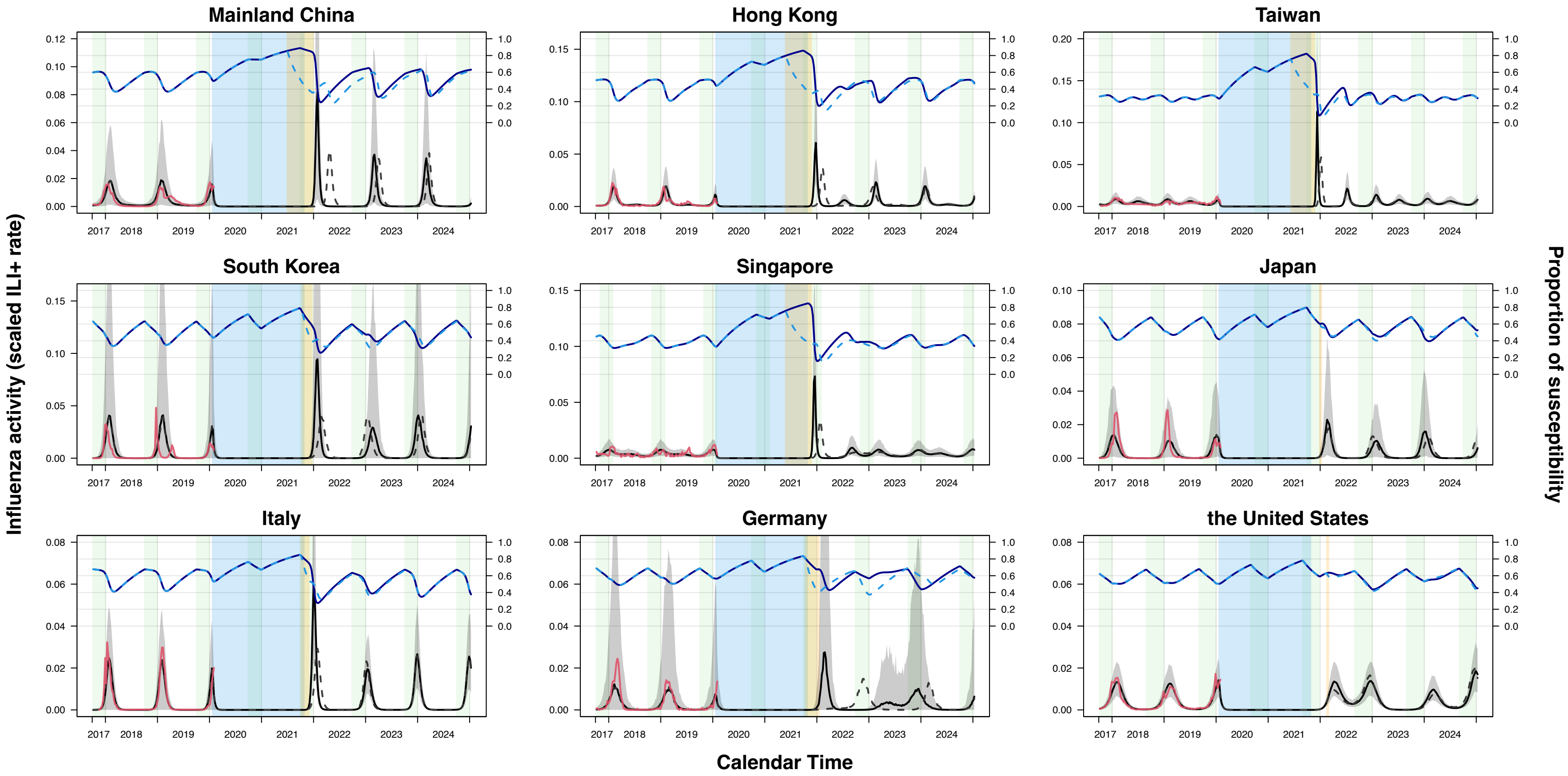

Figure S5: **Prediction of retrospective influenza activity when COVID-19 PHSMs are relaxed by October 2021 (before winter season) with proactive vaccination program (one-off vaccination timing, duration and rate) to mitigate the excess influenza activity in these 9 locations and countries.** The forecast of upcoming seasons was evaluated based on the predictive model framework by fitting the SVIRS models on the influenza activity (observed incidence) data from October 2017 to January 2020 and accounting increased susceptibility and the reduced impact of COVID-19 PHSMs on influenza by location/country. The red lines represent the observed incidence rate, and it was substantially suppressed during 2020 season and subsequent seasons due to the impact of COVID-19 PHSMs, indicated by the blue shaded regions for respective locations. The light green shaded regions indicated the routine vaccination periods for respective locations/countries. The solid black lines are the mean forecast (in 1000 simulations) of incidence rate for 2022 summer seasons under no proactive vaccination/ intervention for influenza, with the range of 2.5th and 97.5th quantiles in grey shaded areas. The dashed black lines are the mean forecast of infection rate under a one-off vaccination program to avoid the excess infection burden in the first upcoming season of influenza. The vaccination start timing was set to the day when the incidence rate crossed the pre-defined threshold and the vaccination period (in orange region) was optimized to achieve the baseline activity (as was in 2017-2019 seasons) with projected vaccination rate of 0.05 per week of total susceptible population. The solid blue lines and dashed blue lines are the proportions of susceptible population with and without vaccination respectively. We carried out the forecasting for the successive seasons until December 2024.

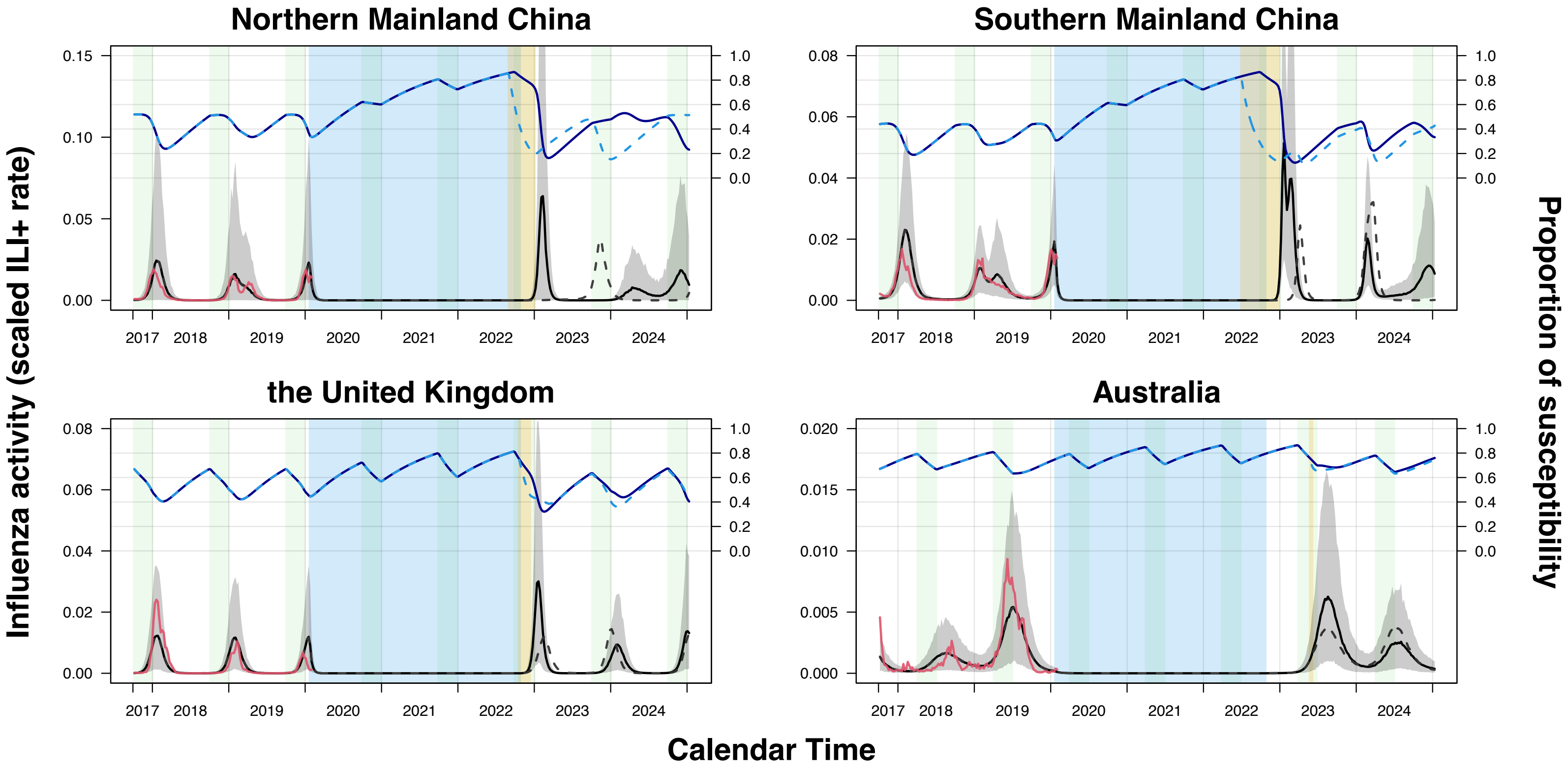

Figure S6. Prediction of upcoming influenza activity when COVID-19 PHSMs are relaxed by October 2022 with proactive vaccination program (one-off vaccination timing, duration and rate) to avoid the excess influenza activity 2020 for the Northern and Southern mainland China, the United Kingdom and Australia (as a country from southern hemisphere). The forecast of upcoming seasons was evaluated based on the predictive model framework by fitting the SVIRS models on the influenza activity (observed incidence) data from October 2017 to January 2020 and accounting increased susceptibility and the reduced impact of COVID-19 PHSMs on influenza by location/country. The red lines represent the observed incidence rate, and it was substantially suppressed during 2020 season and subsequent seasons due to the impact of COVID-19 PHSMs, indicated by the blue shaded regions for respective locations. The green shaded regions indicated the routine vaccination periods for respective locations. The solid black lines are the mean forecast (in 1000 simulations) of incidence rate for 2022 seasons under no proactive vaccination/ intervention for influenza, with the range of 2.5th and 97.5th quantiles in grey shaded areas. The dashed black lines are the mean forecast of infection rate under a one-off vaccination program to avoid the excess infection burden in the first upcoming season of influenza. The vaccination start timing was set to the day when the incidence rate crossed the pre-defined threshold and the vaccination period (in orange region) was optimized to achieve the baseline activity (as was in 2017-2019 seasons) with projected vaccination rate of 0.05 per week of total susceptible population. The solid blue lines and dashed blue lines are the proportions of susceptible population with and without vaccination respectively. We carried out the forecasting for the successive seasons until December 2024. Note: The influenza activity in the United Kingdom started declining from late December 2019 whereas the countrywide COVID-19 PHSMs were implemented much later.

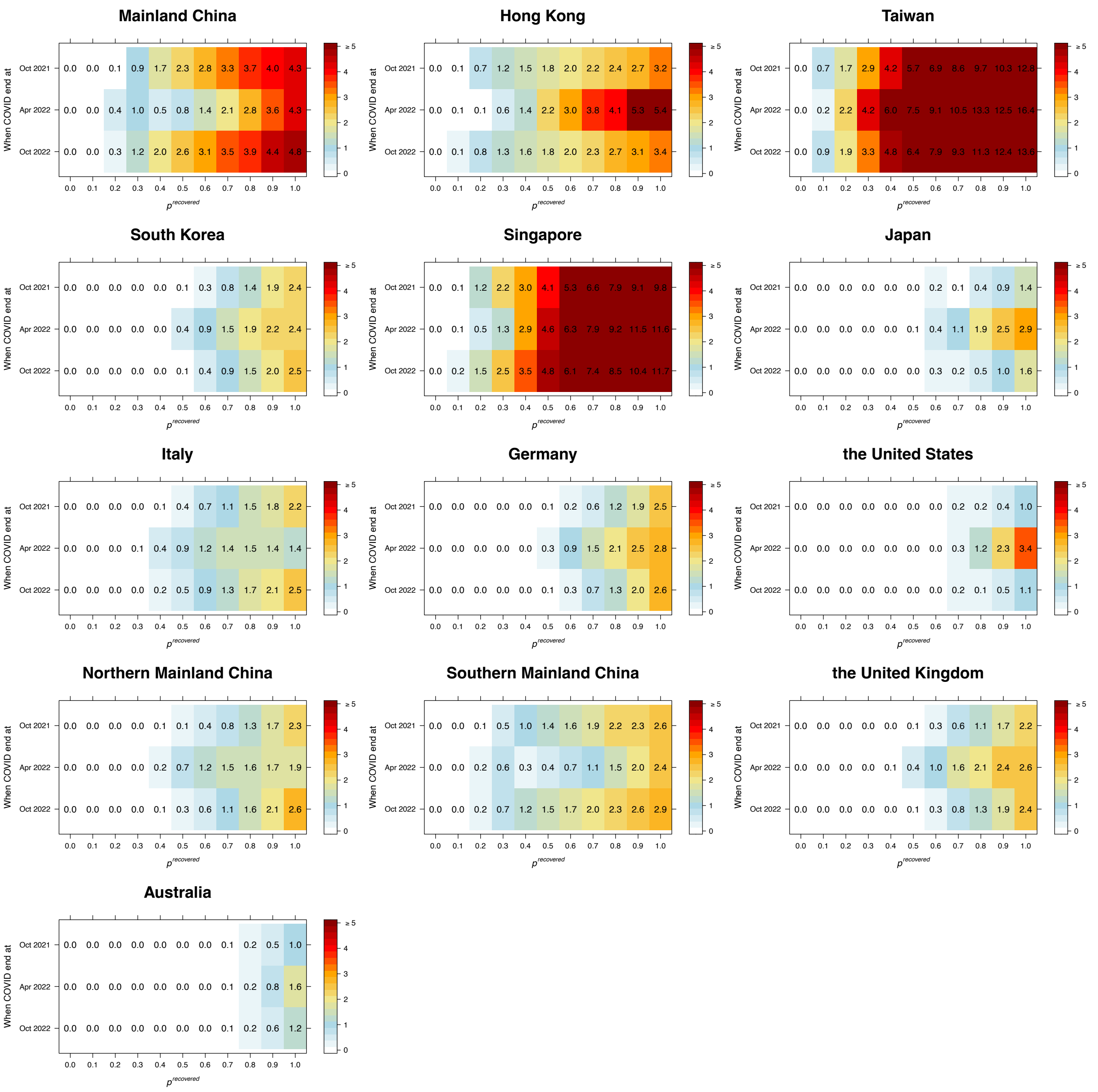

Figure S7. The predicted δ-*fold rise* in '*peak magnitude*' for excess burden in the respective upcoming and retrospective influenza seasons by location/country after COVID-19 PHSMs relaxation under different degrees of recovered transmissibility, reproduction number ($p^{recovered}$). The $p^{recovered}$ is the measure at what level the impact of PHSMs were relaxed. $p^{recovered} = 0$ indicates the no relaxation in PHSMs which keeps the time-varying reproduction number at a very low level so that there are no sustained infections in the community. While $p^{recovered} = 1$ indicates the COVID-19 PHSMs are fully relaxed and the time-varying reproduction number is raised to the level of the usual season start allowing community transmission. The δ-*fold rise* $= 1$ indicates the no excess burden in '*peak magnitude*' and the burden increased or decreased of δ-fold, if $\delta > 1$ or $\delta < 1$ respectively.

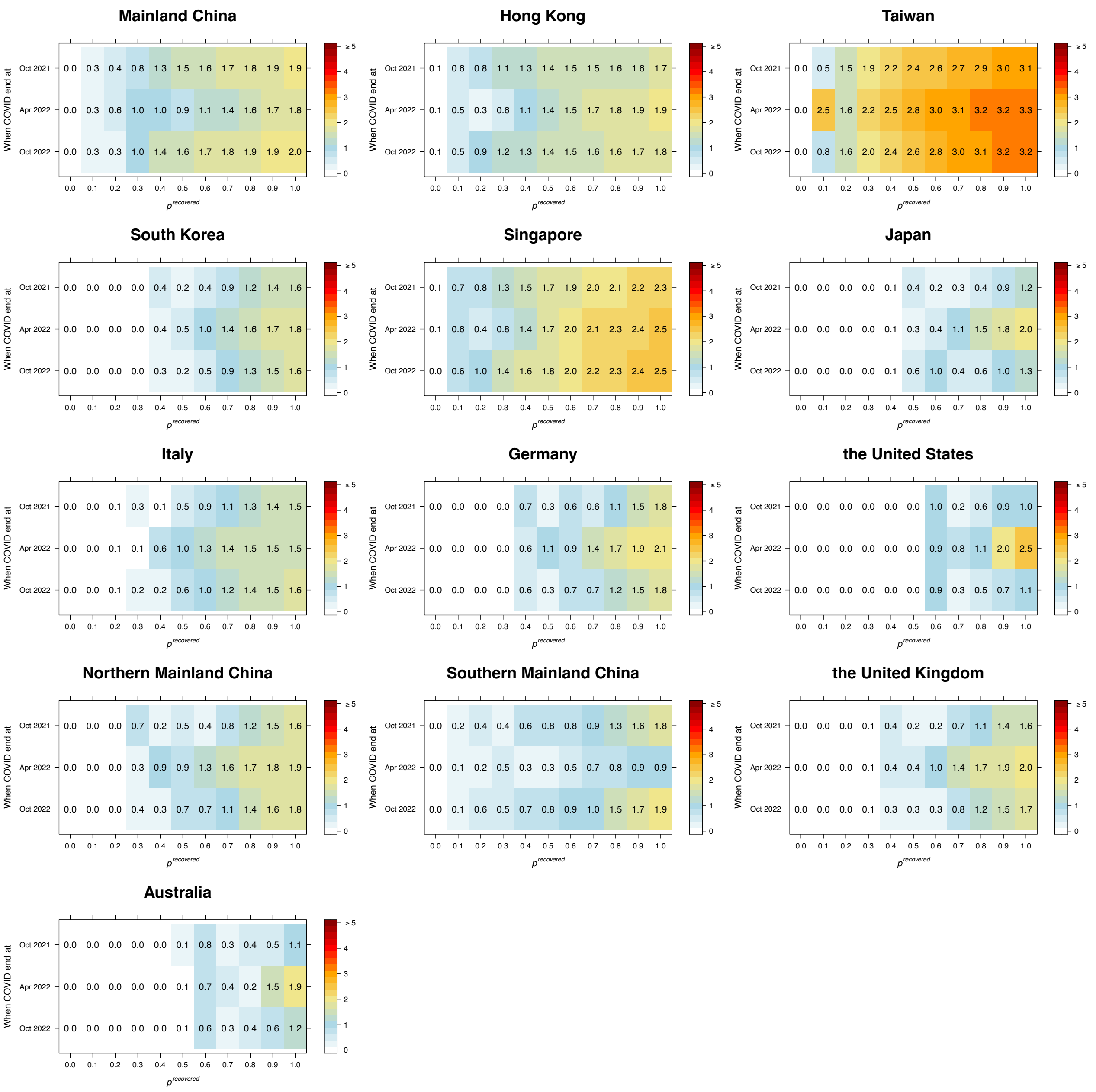

Figure S8. The predicted *δ-fold rise* in '*epidemic size* for excess burden in the respective upcoming and retrospective influenza seasons by location/country after COVID-19 PHSMs relaxation under different degrees of recovered transmissibility, reproduction number ($p^{recovered}$). The $p^{recovered}$ is the measure at what level the impact of PHSMs were relaxed. $p^{recovered} = 0$ indicates the no relaxation in PHSMs which keeps the time-varying reproduction number at very low level so that there are no sustained infections in the community. While $p^{recovered} = 1$ indicates the COVID-19 PHSMs are fully relaxed and the time-varying reproduction number is raised to the level of the usual season start allowing community transmission. The *δ-fold rise* $= 1$ indicates the no excess burden in '*epidemic size'* and the burden increased or decreased of δ-fold, if $\delta > 1$ or $\delta < 1$ respectively.

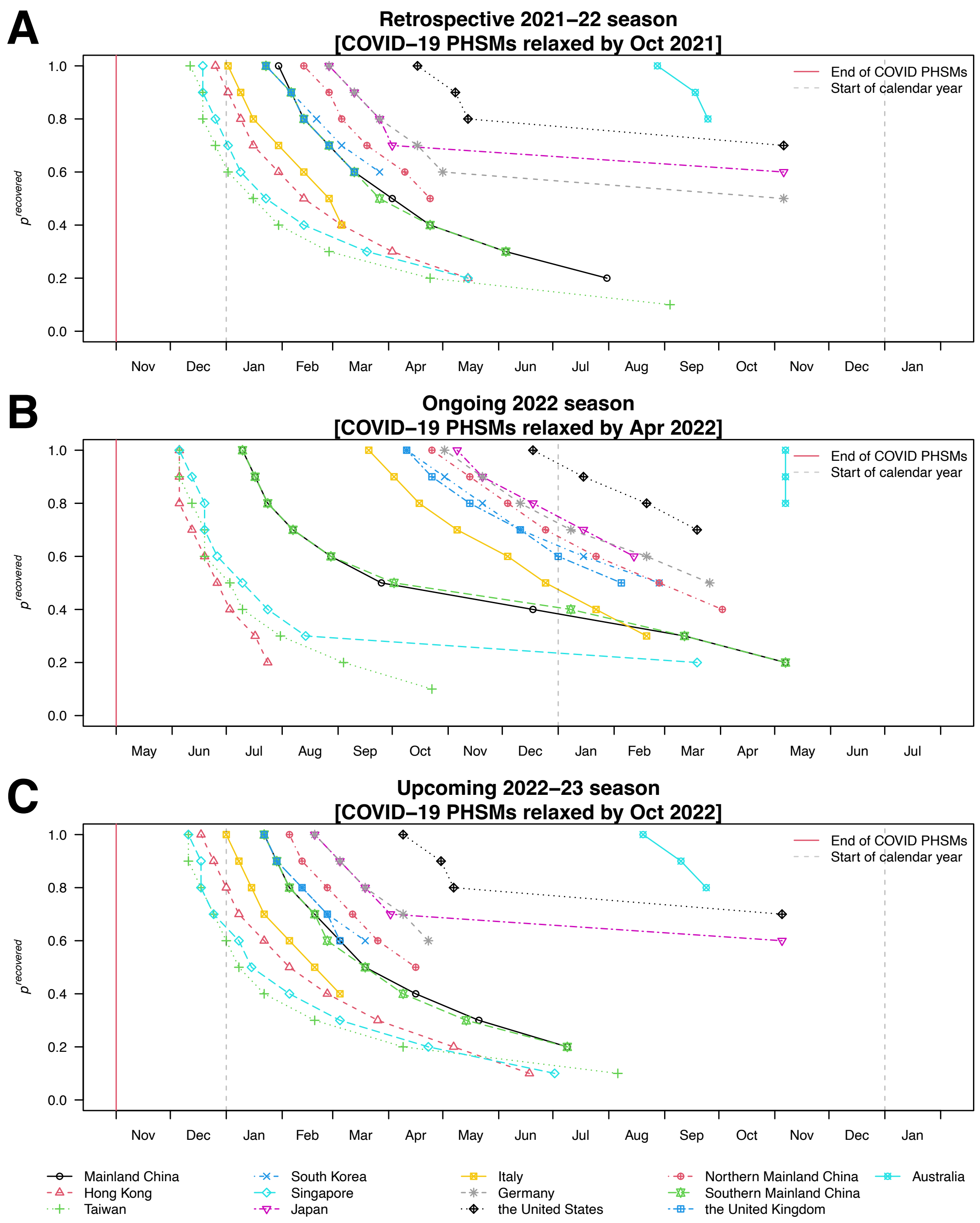

Figure S9. Predicted peak timing of the first influenza epidemics observed within a year after COVID-19 PHSMs relaxation (with different timings and stringency) under different degree of recovered transmissibility, reproduction number ($p^{recovered}$) by location/country. A) Scenario when COVID-19 PHSMs were relaxed in October 2021; B) Scenario when COVID PHSMs were relaxed in April 2022; C) Scenario when COVID PHSMs were relaxed in October 2022. The $p^{recovered}$ is the measure of at what level the impact of PHSMs was relaxed. $p^{recovered} = 0$ indicates the no relaxation in PHSMs which keeps the time-varying reproduction number at a very low level so that there are no sustained infections in the community. While $p^{recovered} = 1$ indicates the PHSMs are fully relaxed and the time-varying reproduction number is raised to the level of the usual season start allowing community transmission.

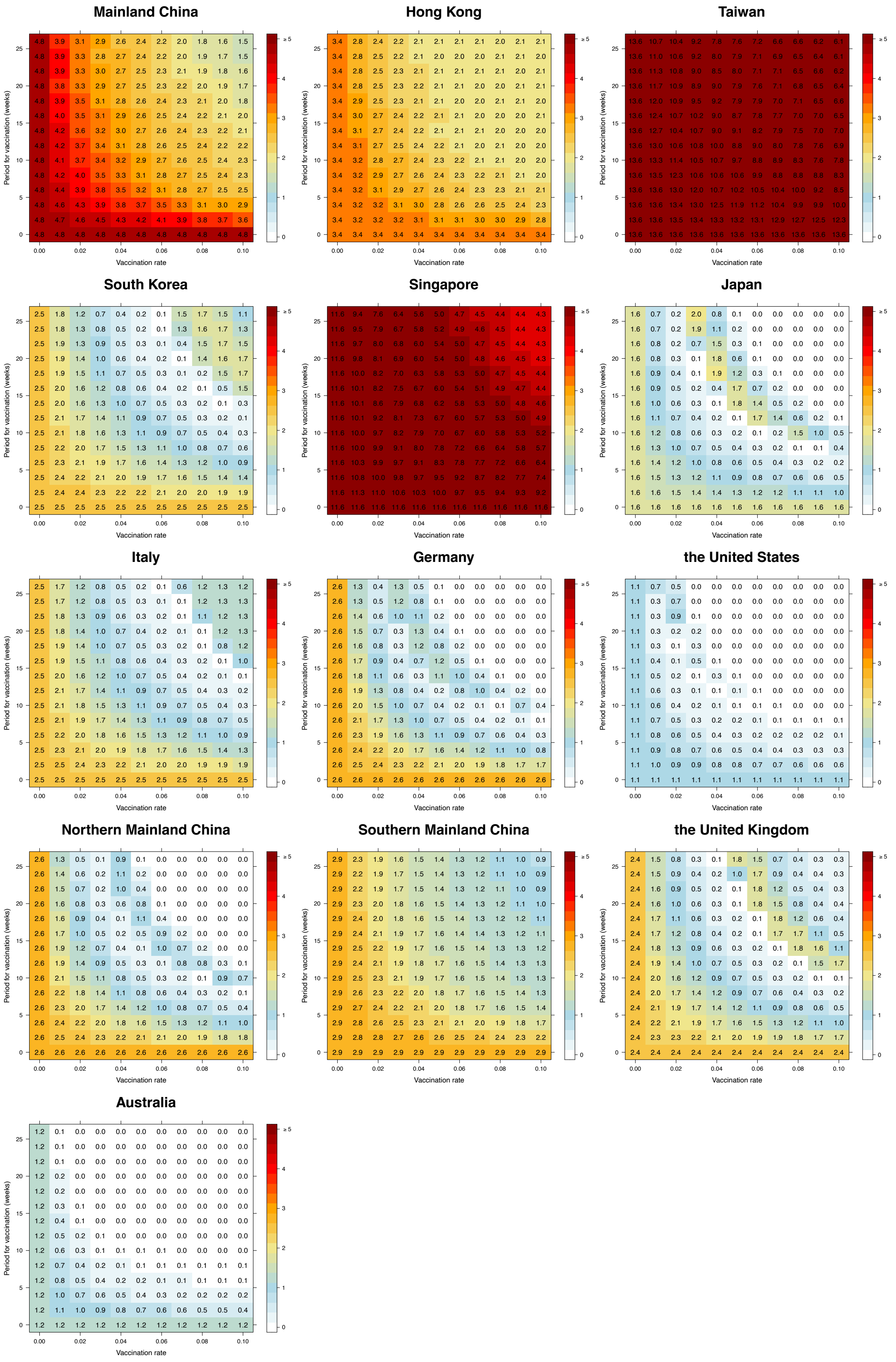

Figure S10. **The predicted** ***δ-fold rise*** **in '*****peak magnitude*****' for excess burden in the upcoming 2022-23 influenza seasons by location/country after COVID-19 PHSMs relaxation (by October 2022) under different possible choices of proactive influenza vaccination rate** (τ_o) **and duration** (τ_o^L)**.** To attain no excess burden in '*peak magnitude*' for these seasons, one should opt the realistic combination of vaccination rate and duration to δ=1.

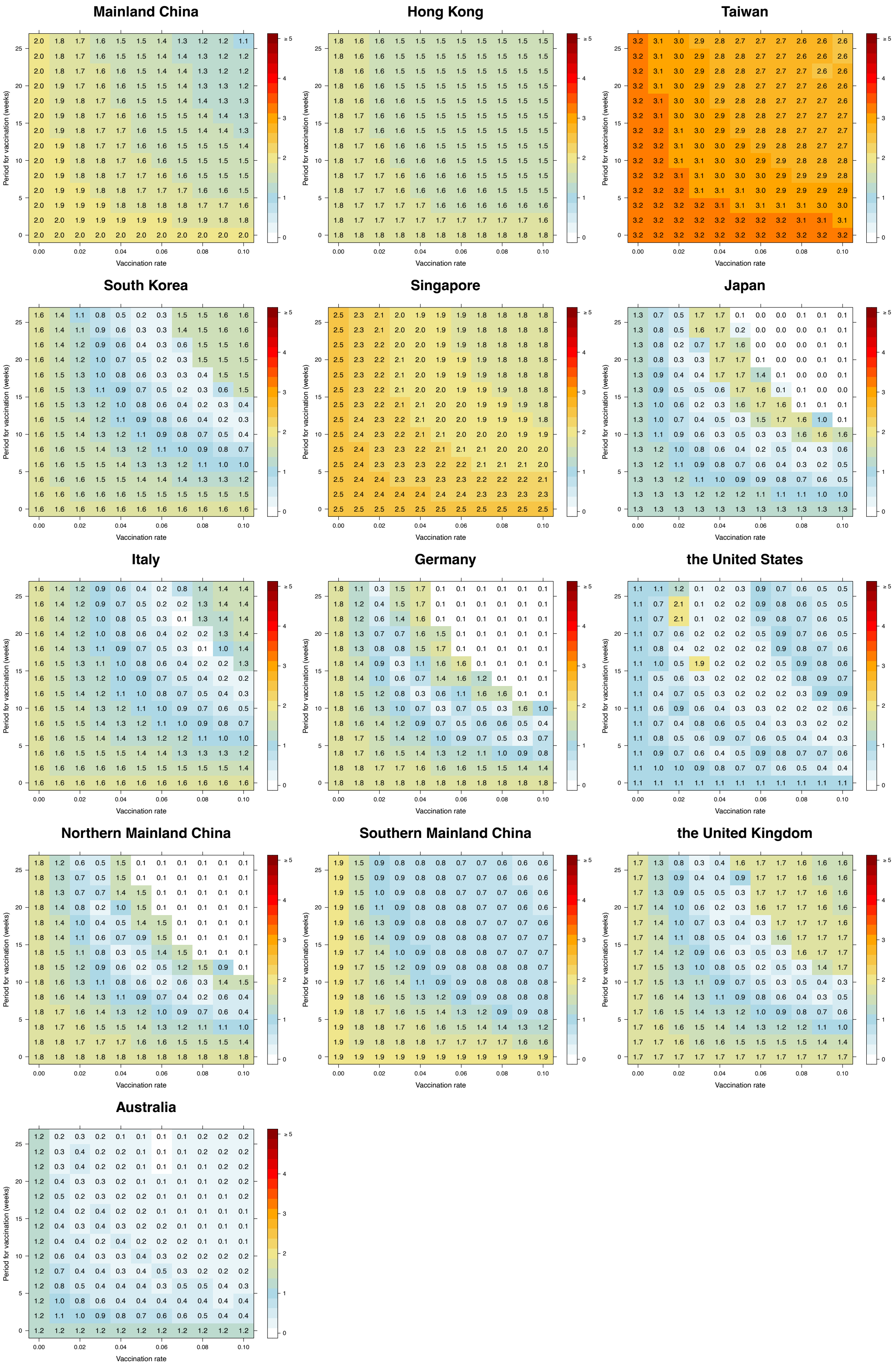

Figure S11. **The predicted** ***δ-fold rise*** **in '*epidemic size*' for excess burden in the upcoming 2022-23 influenza seasons by location/country after COVID-19 PHSMs relaxation (by October 2022) under different possible choices of proactive influenza vaccination rate (τ_o) and duration (τ_o^L).** To attain no excess burden in '*epidemic size*' for these seasons, one should opt for the realistic combination of vaccination rate and duration to δ=1.

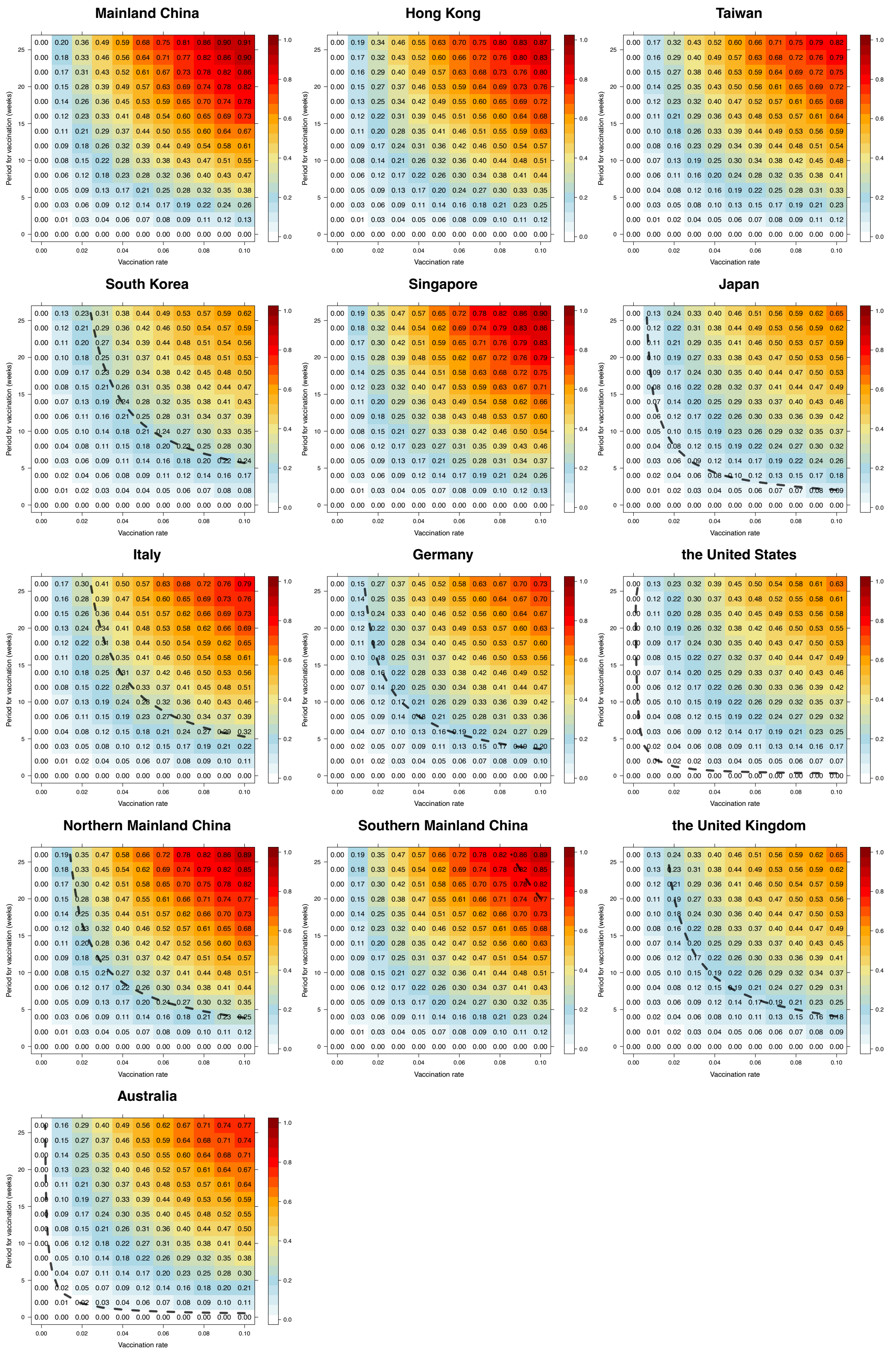

Figure S12. **Additional proportion of vaccinated individual over the population required to alleviate the excess infection burden for the upcoming 2022-23 influenza seasons by location/country after COVID-19 PHSMs relaxation (by October 2022) under the different possible choices of proactive influenza vaccination rate (τ_o) and duration (τ_o^L).** The dashed line indicated the additional minimum proportion of individuals to be vaccinated to achieve the predicted *δ-fold rise* in '*peak magnitude*' = 1 (as illustrated in Figure S10).

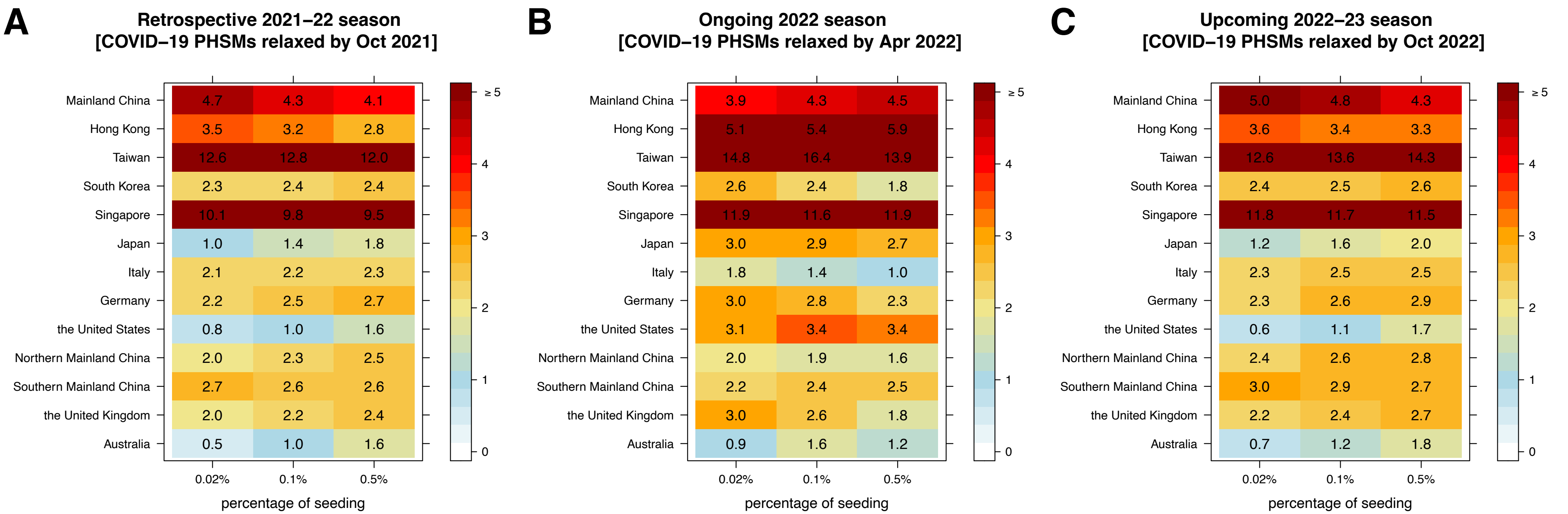

A	0.02%	0.1%	0.5%
Mainland China	4.7	4.3	4.1
Hong Kong	3.5	3.2	2.8
Taiwan	12.6	12.8	12.0
South Korea	2.3	2.4	2.4
Singapore	10.1	9.8	9.5
Japan	1.0	1.4	1.8
Italy	2.1	2.2	2.3
Germany	2.2	2.5	2.7
the United States	0.8	1.0	1.6
Northern Mainland China	2.0	2.3	2.5
Southern Mainland China	2.7	2.6	2.6
the United Kingdom	2.0	2.2	2.4
Australia	0.5	1.0	1.6

B	0.02%	0.1%	0.5%
Mainland China	3.9	4.3	4.5
Hong Kong	5.1	5.4	5.9
Taiwan	14.8	16.4	13.9
South Korea	2.6	2.4	1.8
Singapore	11.9	11.6	11.9
Japan	3.0	2.9	2.7
Italy	1.8	1.4	1.0
Germany	3.0	2.8	2.3
the United States	3.1	3.4	3.4
Northern Mainland China	2.0	1.9	1.6
Southern Mainland China	2.2	2.4	2.5
the United Kingdom	3.0	2.6	1.8
Australia	0.9	1.6	1.2

C	0.02%	0.1%	0.5%
Mainland China	5.0	4.8	4.3
Hong Kong	3.6	3.4	3.3
Taiwan	12.6	13.6	14.3
South Korea	2.4	2.5	2.6
Singapore	11.8	11.7	11.5
Japan	1.2	1.6	2.0
Italy	2.3	2.5	2.5
Germany	2.3	2.6	2.9
the United States	0.6	1.1	1.7
Northern Mainland China	2.4	2.6	2.8
Southern Mainland China	3.0	2.9	2.7
the United Kingdom	2.2	2.4	2.7
Australia	0.7	1.2	1.8

Figure S13. The predicted *δ-fold rise* in '*peak magnitude*' under different percentage of seeding (for given the transmissibility is fully recovered) presented by location/country. A) Scenario when COVID-19 PHSMs were relaxed in October 2021; B) Scenario when COVID PHSMs were relaxed in April 2022; C) Scenario when COVID PHSMs would be relaxed in October 2022.

8. Tables S1 to S7

Table S1: The timing of the public health social measures implemented in response of COVID-19 in different locations/ countries including mainland China, Hong Kong, Taiwan, South Korea, Singapore, Japan, Italy, Germany, the United Kingdom (UK) and the United States of America (USA) specifically during 2020. These PHSMs are classified in to case-based, community-wide and travel-realted control measures and available as Appendix 2 online at https://doi.org/10.6084/m9.figshare.18517028, at figshare [24].

Table S2: The timing, duration and coverage of seasonal influenza vaccination program (routing annual vaccination program) in the studied countries/ locations during 2017-2022. We retrieved the information from the available governments/media reports and literatures. * For the countries/ locations with missing (or unavailable) information on the timing and duration, we considered the routine vaccination period as October – December. † While some of the countries/ location had information on the start of such vaccination program, but the end timings were not clearly identified from the report or literation therefore, we restrict the duration for maximum of 3 months (~13 weeks) for such countries/ locations for each season.

Countries/ Locations	Vaccination coverage	Frequency of Vaccination per Year	Vaccination Period	Remarks and References
Mainland China	9.4%	Once a Year	October-December *	**Coverage:** 9.4% (as per the data during 2018), (Wang, Q. et al, Influenza vaccination coverage of population and the factors influencing influenza vaccination in mainland China: A meta-analysis, Vaccine, Volume 36, Issue 48, 2018, Pages 7262-7269, https://doi.org/10.1016/j.vaccine.2018.10.045. https://www.sciencedirect.com/science/article/pii/S0264410X18314117?via%3Dihub) **Timing & Duration:** Note: No scientific information on the national level flu seasonal vaccination timing and duration are available. Therefore, we assumed the timing of vaccination for mainland China.
Hong Kong	17.0%	Once a Year	October-December †	**Coverage:** Data from 2019 to 27/2/2022, 17.6% (19-20 season); 17.24% (20-21 season); 15.78% (21-22 season) (https://www.chp.gov.hk/en/features/102226.html;

				Centre for Health Protection - Frequently Asked Questions on Seasonal Influenza Vaccination for the 2021/22 Season in Hong Kong (chp.gov.hk)).<br>**Timing & Duration:** Starts from 9th October 2019, 8th October 2020, 6th October 2021 for respective season. But no clear information for the end of the vaccination program, we assumed December as the end of vaccination for each season.<br>(2019/20 seasonal influenza vaccination programmes to be launched in October (info.gov.hk); 2020/21 seasonal influenza vaccination programmes to launch in October (info.gov.hk);<br>Centre for Health Protection - Government Vaccination Programme (GVP) 2021/22 (chp.gov.hk))
Taiwan	25.0%	Once a Year	October - December	**Coverage:** During 2016-2017 data. 25% (in 2016 flu season), 25% (in 2017 flu season))<br>(Meyer D, Shearer MP, Chih YC, Hsu YC, Lin YC, Nuzzo JB. Taiwan's Annual Seasonal Influenza Mass Vaccination Program-Lessons for Pandemic Planning. Am J Public Health. 2018;108(S3):S188-S193. doi:10.2105/AJPH.2018.304527, https://www.ncbi.nlm.nih.gov/pmc/articles/PMC6129648/)<br>**Timing & Duration:** In 2016, on 1st October the government-funded seasonal influenza (flu) vaccination campaign was launched and by 1 December 2016 it is expected the vaccine will be depleted in some cities and counties within 1 to 2 weeks' time.<br>(Beginning December 1, 2016, government-funded flu vaccine available for all citizens and Taiwan CDC to expand target population for government-funded influenza antiviral drugs - 衛生福利部疾病管制署;<br>https://www.cdc.gov.tw/En/Bulletin/Detail/oLvhnLRfc-HlcCLHKfkexQ?typeid=158)
South Korea	44.1%	Once a Year	October – December *	**Coverage:** As for 2014 data, the total influenza vaccination coverage increased from 38.0% in 2005 to 44.1% in 2014. (Jeongmin Seo, Juwon Lim, Trends in influenza vaccination coverage rates in South Korea from 2005 to 2014: Effect of public health policies on vaccination behavior, Vaccine, Volume 36, Issue 25, 2018, Pages 3666-3673, https://www.sciencedirect.com/science/article/pii/S0264410X18306315?via%3Dihub<br>vaccines-09-00367-v2.pdf;<br>Hyeongap Jang & Joon Hyung Kim (2019) Factors affecting influenzavaccination in adults aged 50-64 years with high-risk chronic diseases in South Korea, HumanVaccines & Immunotherapeutics, 15:4, 959-966, DOI: 10.1080/21645515.2018.1556075)

				**Timing & Duration:** Note: No scientific information on the national level flu seasonal vaccination timing and duration are available. Therefore, we assumed the timing of vaccination for South Korea.
Singapore	20%	Once a Year	November - January †	**Coverage:** We could only retrieve the information on the vaccination coverage for Singapore in recent years as 24% for the age-groups 65- 79 in 2019. We assumed 20% for overall population. (https://www.moh.gov.sg/news-highlights/details/adult-immunisation-vaccinations-take-up-rate Influenza Vaccine - Clari Health SG) **Timing & Duration:** (Hanley JH et al, 2019: Increasing Influenza and Pneumococcal Vaccination Uptake in Seniors Using Point-of-Care Informational Interventions in Primary Care in Singapore: A Pragmatic, Cluster-Randomized Crossover Trial American Journal of Public Health 109, 1776_1783, https://doi.org/10.2105/AJPH.2019.305328)
Japan	40%	Once a Year	October - December	**Coverage:** In recent years, the influenza vaccination rate was only approximately 40% with no much change year wise. (Watanabe, D., Harada, T. and Hiroshige, J. (2021), Changes in influenza vaccination coverage associated during the COVID-19 pandemic in Japan. J Gen Fam Med, 22: 359-360. https://doi.org/10.1002/jgf2.462 Hiroaki N, Yumi W, Yoshihiko M. Estimation of influenza vaccination coverage in Japan. Nihon Koushueisei Zassi. 2014; 61(7):354–9.(In Japanese). Kajikawa N. et al.　Factors associated with influenza vaccination in Japanese elderly outpatients. Infection, Disease & Health, 2019, Volume 24, Issue 4, 212 – 221. https://doi.org/10.1016/j.idh.2019.07.002) **Timing & Duration:** Influenza vaccination effectiveness for people aged under 65 years in Japan, 2013/2014 season: application of a doubly robust method to a large-scale, real-world dataset - PMC (nih.gov); Q&A on Influenza｜厚生労働省 (mhlw.go.jp)).

Italy	16% before 2019-20 season<br>24% since 2020-21 season	Once a year	October – December †	**Coverage:** As per the data during 2017- 2022, the vaccination coverage was 15.3 (in 2012-18 season), 15.8% (in 2018-19 season) 16.8% (in 2019-20 season) and 23. 7 (in 2020-21 season).<br>(Pitrelli A. Introduction of a quadrivalent influenza vaccine in Italy: a budget impact analysis. J Prev Med Hyg. 2016;57(1):E34-E40; Italy: coverage rate of flu vaccination 2021 | Statista).<br>**Timing & Duration:** The end of the routine vaccination scheme were not clearly identified from the report or literation therefore, we restrict the duration for Italy for maximum of 3 months (12-13 weeks) for each season.<br>(Flu Shot In Italy: Everything You Need To Know In 2020 | Doctors In Italy; https://www.thelocal.it/20201015/everything-you-need-to-know-about-getting-a-flu-vaccination-in-italy/)
Germany	30%	Once a year	October – December †	**Coverage:** Using 2016-19 data we could retrieve the vaccination coverage for Germany were 39.5%, 39.3% for health care workers in 2016/2017 and 2017/2018 and 39% for age 65 or over in 2019 only. Therefore, we assumed the overall coverage was 30%. (Julia Neufeind, Ronja Wenchel, Birte Boedeker, Sabine Wicker & Ole Wichmann (2021) Monitoring influenza vaccination coverage and acceptance among health-care workers in German hospitals – results from three seasons, Human Vaccines & Immunotherapeutics, 17:3, 664-672, DOI: 10.1080/21645515.2020.180107.<br>Influenza: Vaccination rates in international comparison - German Federal Statistical Office (destatis.de))<br>**Timing & Duration:** Flu vaccination | SBK
The United States	45%	Once a Year	September – December	**Coverage:** Based on Recent Data, we could retrieve the vaccination coverage of 37.1% - 50.2% in 2017/18 – 2020/21 seasons; 44.3% as of 29/1/2022 for all adults age > 18. Therefore, we consider the overall population level coverage as around 45%.<br>(https://www.cdc.gov/flu/fluvaxview/coverage-2021estimates.htm#:~:text=Overall%2C%20among%20adults;<br>Latest U.S. Flu Vaccination Coverage Estimates Continue to Show Concerning Drops in Coverage Among People at Increased Risk for Flu Complications | CDC;<br>Adult Immunization Schedule by Vaccine and Age Group | CDC)<br>**Timing & Duration:** Key Facts About Seasonal Flu Vaccine | CDC.

The United Kingdom	40%	Once a Year	October – December †	**Coverage:** In recent years in England, the vaccination coverage was of 48% for clinical at-risk group aged 16-64; 70.5%- 82% from 2016/17 -2021/22 for age 65 or over. No temporal information on vaccination coverage is available. We assume 40% for overall population.<br>(Adult flu vaccination coverage | The Nuffield Trust;<br>Inactivated Flu Vaccine | Vaccine Knowledge (ox.ac.uk))<br>**Timing & Duration:** (The national influenza immunisation programme 2021 to 2022: August 2021 (publishing.service.gov.uk)<br>https://pubmed.ncbi.nlm.nih.gov/34560879/).
Australia	26%	Once a Year	April – June †	**Coverage:** As per the 2014 data, we calculated the vaccination coverage for Australia was around 26% each year. (https://www.cambridge.org/core/journals/epidemiology-and-infection/article/pooled-influenza-vaccine-effectiveness-estimates-for-australia-20122014/5FDC2D885A8AF020C952F47395AE09B8;<br>Influenza (flu) | The Australian Immunisation Handbook (health.gov.au);<br>Flu vaccine FAQs | healthdirect.)<br>**Timing & Duration:** Generally, the new season influenza vaccines under were expected to be available from April for each year, but no clear information of the duration. We assumed it to be around 3 months.<br>(Influenza (flu) vaccine | Australian Government Department of Health)

Table S3: Yearly number of visits in the studied countries/ locations during 2017-2022. We retrieved the information from the available reports or websites of respective governments and travel agencies.

Countries / Locations	Yearly visits (Millions)	Comments & References
Mainland China	135.1	https://www.travelchinaguide.com/tourism/; http://www.stats.gov.cn/tjsj/ndsj/2011/indexeh.htm
Hong Kong	60	https://www.tourism.gov.hk/en/tourism-statistics-2018.php; https://www.info.gov.hk/gia/general/201802/13/P2018021300299.htm
Taiwan	11.9	https://admin.taiwan.net.tw/; https://eng.stat.gov.tw/lp.asp?CtNode=6339&CtUnit=1072&BaseDSD=36&mp=5
South Korea	17.5	https://kto.visitkorea.or.kr/eng.kto; https://web.archive.org/web/20110506065230/http://esa.un.org/unpd/wpp/index.htm
Singapore	18.5	https://web.archive.org/web/20170122112656/ https://www.stb.gov.sg/statistics-and-market-insights/Pages/statistics-Visitor-Arrivals.aspx; https://www.population.gov.sg/our-population/population-trends/overview
Japan	31.9	https://statistics.jnto.go.jp/en/graph/#graph--inbound--travelers--transition; https://www.stat.go.jp/english/data/jinsui/2017np/index.html#a15k28-a
Italy	128.1	https://www.istat.it/it/files//2019/12/Asi-2019.pdf; https://unstats.un.org/unsd/demographic/products/dyb/dybsets/1948%20DYB.pdf
Germany	35.6	https://www.destatis.de/DE/Home/_inhalt.html
The United States	70.1	https://www.dhs.gov/immigration-statistics/yearbook/2015/table28; https://www.cia.gov/the-world-factbook/
The United Kingdom	140	https://www.gov.uk/government/statistics/immigration-statistics-year-ending-december-2021/how-many-people-come-to-the-uk-each-year-including-visitors; https://www.ons.gov.uk/peoplepopulationandcommunity/populationandmigration/populationestimates/timeseries/ukpop/pop
Australia	8.6	https://www.abs.gov.au/AUSSTATS/abs@.nsf/Previousproducts/3401.0Feature%20Article1Dec%202010?opendocument&tabname=Summary&prodno=3401.0&issue=Dec%202010&num=&view=; https://www.abs.gov.au/statistics/people/population/national-state-and-territory-population/latest-release

Table S4: Description of model parameters and variables with their mode of estimation under the scheme of mechanistic model as illustrated in Figure S3.

Model variables and parameters	Description	Values and mode of estimation
$S(t)$	Susceptible individuals at time t	Estimated by model
$V(t)$	Vaccinated individuals at time t	Estimated by model
$I(t)$	Infectious individuals at time t	Estimated by model
$R(t)$	Recovered individuals at time t	Estimated by model
θ and μ	Birth rate and death rate	$\theta = \mu$ = 1/80 per year (Assumed)
$\beta_e(t)$	Transmission rate	Estimated by model
$\tau(t)$	Vaccination rate	Refer Table S2 (Literature and sensitivity analysis)
ω	Vaccine efficacy	0.59 (Literature [38])
$1/\gamma$	Mean infectious period	4 days (Literature [45])
$1/\rho_R$	Mean infection-acquired immunity waning delay	1 year (Assumed)
$1/\rho_V$	Mean vaccine-acquired immunity waning delay	9 months (Literature [36])
$\epsilon(t)$	Seeding of infectious individuals	Refer Table S3 (Literature and sensitivity analysis)
σ	Overdispersion for fitting	Estimated by model
ϕ	Scaling factor for fitting	Estimated by model

Table S5: Predicted reduction in attack rate or infections via simulation framework with different choices of pre-immunity in the population (sensitivity analysis).

Locations/ Countries	Parameters $(\sigma,\ \gamma,\ E_o,\ I_o)$	% of Pre-existing immunity	S_0	R_0	% reduction in infections (95% CI)
Mainland China	(0.50, 0.59, 0.0001, 0.0001)	0.1	0.999	1.30 (1.18, 1.44)	10.26 (2.86,14.2)
		10.1	0.899	1.45 (1.31, 1.61)	9.36 (2.62,12.95)
		20.1	0.799	1.63 (1.47, 1.81)	8.43 (2.37,11.66)
		30.1	0.699	1.86 (1.68, 2.06)	7.46 (2.11,10.31)
Hong Kong	(0.50, 0.50, 0.0001, 0.0001)	0.1	0.999	1.45 (1.29, 1.64)	21.02 (10.66, 26.65)
		10.1	0.899	1.61 (1.43, 1.82)	19.43 (9.88, 24.66)
		20.1	0.799	1.81 (1.61, 2.05)	17.74 (9.05, 22.54)
		30.1	0.699	2.07 (1.84, 2.34)	15.95 (8.16, 20.27)
Taiwan	(0.50, 0.50, 0.0001, 0.0001)	0.1	0.999	1.28 (1.12, 1.45)	14.31 (11.70, 16.11)
		10.1	0.899	1.42 (1.24, 1.62)	13.05 (10.67, 14.70)
		20.1	0.799	1.59 (1.40, 1.82)	11.75 (9.60, 13.24)
		30.1	0.699	1.82 (1.60, 2.08)	10.39 (8.49, 11.72)
South Korea	(0.50, 0.56, 0.0001, 0.0001)	0.1	0.999	1.30 (1.23, 1.37)	5.08 (1.45, 7.21)
		10.1	0.899	1.44 (1.37, 1.52)	4.59 (1.31, 6.52)
		20.1	0.799	1.63 (1.54, 1.71)	4.09 (1.17, 5.82)
		30.1	0.699	1.86 (1.76, 1.96)	3.59 (1.03, 5.10)
Singapore	(0.53, 0.50, 0.0001, 0.0001)	0.1	0.999	1.25 (1.14, 1.37)	24.8 (20.82, 27.51)
		10.1	0.899	1.39 (1.26, 1.53)	22.94 (19.25, 25.46)
		20.1	0.799	1.56 (1.42, 1.72)	20.95 (17.59, 23.28)
		30.1	0.699	1.79 (1.62, 1.96)	18.84 (15.81, 20.94)
Japan	(0.50, 0.56, 0.0001, 0.0001)	0.1	0.999	1.29 (1.25, 1.34)	7.57 (3.72, 10.34)
		10.1	0.899	1.44 (1.39, 1.49)	6.91 (3.40, 9.45)
		20.1	0.799	1.62 (1.56, 1.67)	6.23 (3.06, 8.52)
		30.1	0.699	1.85 (1.79, 1.91)	5.53 (2.72, 7.56)
Italy	(0.50, 0.50, 0.0001, 0.0001)	0.1	0.999	1.24 (1.20, 1.28)	13.66 (12.46, 14.70)
		10.1	0.899	1.38 (1.33, 1.42)	12.49 (11.39, 13.45)
		20.1	0.799	1.55 (1.50, 1.60)	11.27 (10.27, 12.14)
		30.1	0.699	1.77 (1.72, 1.83)	10.96 (10.10, 11.61)
Germany	(0.50, 0.50, 0.0001, 0.0001)	0.1	0.999	1.17 (1.08, 1.26)	10.96 (10.1, 11.61)
		10.1	0.899	1.30 (1.20, 1.40)	9.91 (9.13, 10.51)
		20.1	0.799	1.46 (1.36, 1.57)	8.85 (8.14, 9.38)
		30.1	0.699	1.67 (1.55, 1.80)	7.75 (7.13, 8.22)
The USA	(0.50, 0.56, 0.0001, 0.0001)	0.1	0.999	1.15 (1.06, 1.24)	7.71 (7.50, 7.87)
		10.1	0.899	1.28 (1.18,1.38)	6.97 (6.78, 7.11)
		20.1	0.799	1.44 (1.33,1.55)	6.20 (6.04, 6.34)
		30.1	0.699	1.64 (1.52,1.78)	5.43 (5.28, 5.54)

Table S6: Parameter estimates and their 95% profile confidence interval of predictive mechanistic models.

Locations/ countries	α_0	α_1	α_2	σ	ϕ
**Mainland China**	-0.55 (-0.81, -0.27)	-0.47 (-0.69, -0.34)	0.05 (-0.01, 0.11)	1.38 (1.06, 1.78)	0.43 (0.33, 0.65)
**Hong Kong**	0.26 (0.21, 0.41)	-0.96 (-1.11, -0.93)	-0.11 (-0.15, -0.06)	5.65 (3.94, 6.08)	0.31 (0.30, 0.38)
**Taiwan**	5.03 (3.68, 6.97)	-3.37 (-5.38, -1.88)	-1.44 (-1.97, -1.07)	6.43 (4.59, 7.97)	0.31 (0.29, 0.35)
**South Korea**	-0.99 (-1.26, -0.72)	0.13 (-0.01, 0.26)	0.80 (0.71, 0.89)	0.42 (0.33, 0.52)	0.90 (0.71, 1.00)
**Singapore**	0.52 (0.14, 1.56)	-0.54 (-1.04, -0.42)	0.06 (-0.14, 0.16)	4.14 (3.27, 5.34)	0.31 (0.28, 0.36)
**Japan**	-1.45 (-1.53, -1.37)	0.30 (0.25, 0.34)	0.73 (0.71, 0.77)	1.44 (1.20, 1.86)	0.49 (0.42, 0.56)
**Italy**	-1.18 (-1.32, -1.04)	0.26 (0.15, 0.36)	0.75 (0.67, 0.85)	6.96 (4.95, 8.77)	0.45 (0.40, 0.51)
**Germany**	-1.58 (-1.88, -0.83)	0.02 (-0.22, 0.18)	0.61 (0.30, 0.72)	0.16 (0.13, 0.20)	0.46 (0.22, 0.91)
**The United States**	-1.75 (-1.88, -1.70)	0.21 (0.18, 0.26)	0.69 (0.64, 0.75)	10.16 (7.82, 12.79)	0.97 (0.93, 1.00)
**The United Kingdom**	-1.32 (-1.45, -0.83)	0.24 (-0.06, 0.29)	0.71 (0.60, 0.74)	1.87 (1.52, 2.46)	0.39 (0.28, 0.44)
**Australia**	-2.93 (-3.06, -2.86)	-0.31 (-0.42, -0.24)	-0.79 (-0.88, -0.69)	2.51 (1.99, 2.68)	0.65 (0.57, 0.75)

Table S7: Estimation of excess burden in terms of δ-fold rise (mean with 2.5^{th} and 97.5^{th} percentile in brackets) in '*peak magnitude*' and '*epidemic size*' for the respective upcoming and retrospective influenza seasons by locations/ countries after COVID-19 PHSMs relaxation.

	2021-22 season (COVID-19 PHSMs relaxed by October 2021)		2022 season (COVID-19 PHSMs relaxed by April 2022)		2022-23 season (COVID-19 PHSMs relaxed by October 2022)	
**Locations/ countries**	δ-fold rise in peak magnitude	δ-fold rise in epidemic size	δ-fold rise in peak magnitude	δ-fold rise in epidemic size	δ-fold rise in peak magnitude	δ-fold rise in epidemic size
**Mainland China**	4.3 (4.2, 4.4)	1.9 (1.9, 1.9)	4.3 (4.3, 4.4)	1.8 (1.8, 1.8)	4.8 (4.8, 4.9)	2.0 (1.9, 2.0)
**Hong Kong**	3.2 (3.0, 3.3)	1.7 (1.6, 1.7)	5.4 (5.2, 5.6)	1.9 (1.9, 2.0)	3.4 (3.2, 3.5)	1.8 (1.7, 1.8)
**Taiwan**	12.8 (12.5, 13.0)	3.1 (2.9, 3.1)	16.4 (16.0, 16.6)	3.3 (3.2, 3.4)	13.6 (12.9, 14.3)	3.2 (3.1, 3.3)
**South Korea**	2.4 (2.3, 2.4)	1.6 (1.6, 1.6)	2.4 (2.3, 2.4)	1.8 (1.7, 1.8)	2.5 (2.5, 2.6)	1.6 (1.6, 1.7)
**Singapore**	9.8 (9.2, 10.4)	2.3 (2.2, 2.4)	11.6 (11.0, 12.3)	2.5 (2.3, 2.6)	11.7 (11.0, 12.2)	2.5 (2.3, 2.6)
**Japan**	1.4 (1.1, 1.7)	1.2 (1.0, 1.4)	2.9 (2.4, 3.4)	2.0 (1.7, 2.2)	1.6 (1.3, 1.9)	1.3 (1.2, 1.5)
**Italy**	2.2 (2.1, 2.4)	1.5 (1.5, 1.6)	1.4 (1.3, 1.4)	1.5 (1.5, 1.6)	2.5 (2.4, 2.6)	1.6 (1.6, 1.7)
**Germany**	2.5 (2.5, 2.5)	1.8 (1.7, 1.8)	2.8 (2.7, 2.8)	2.1 (2.0, 2.1)	2.6 (2.6, 2.7)	1.8 (1.8, 1.9)
**The United States**	1.0 (0.9, 1.1)	1.0 (0.9, 1.1)	3.4 (3.2, 3.6)	2.5 (2.3, 2.7)	1.1 (1.0, 1.2)	1.1 (1.0, 1.2)
**The United Kingdom**	2.2 (2.1, 2.3)	1.6 (1.5, 1.7)	2.6 (2.5, 2.6)	2.0 (1.9, 2.0)	2.4 (2.3, 2.5)	1.7 (1.6, 1.8)
**Australia**	1.0 (0.6, 1.5)	1.1 (0.8, 1.6)	1.6 (1.3, 2.1)	1.9 (1.6, 2.5)	1.2 (0.8, 1.7)	1.2 (0.9, 1.7)

9. References

1. Ali ST, Cowling BJ, Lau EHY, Fang VJ, Leung GM. Mitigation of Influenza B Epidemic with School Closures, Hong Kong, 2018. *Emerg Infect Dis* 2018; **24**(11): 2071-3.
2. Wong JY, Wu P, Nishiura H, et al. Infection fatality risk of the pandemic A(H1N1)2009 virus in Hong Kong. *Am J Epidemiol* 2013; **177**(8): 834-40.
3. Goldstein E, Cobey S, Takahashi S, Miller JC, Lipsitch M. Predicting the epidemic sizes of influenza A/H1N1, A/H3N2, and B: a statistical method. *PLoS Medicine* 2011; **8**(7): e1001051.
4. Yang W, Cowling BJ, Lau EH, Shaman J. Forecasting Influenza Epidemics in Hong Kong. *PLoS Comput Biol* 2015; **11**(7): e1004383.
5. Ali ST, Wu P, Cauchemez S, et al. Ambient ozone and influenza transmissibility in Hong Kong. *Eur Respir J* 2018; **51**(5).
6. Wu P, Presanis AM, Bond HS, Lau EHY, Fang VJ, Cowling BJ. A joint analysis of influenza-associated hospitalizations and mortality in Hong Kong, 1998-2013. *Sci Rep* 2017; **7**(1): 929.
7. Ryu S, Ali ST, Cowling BJ, Lau EHY. Effects of School Holidays on Seasonal Influenza in South Korea, 2014-2016. *J Infect Dis* 2020; **222**(5): 832-5.
8. Kandula S, Yamana T, Pei S, Yang W, Morita H, Shaman J. Evaluation of mechanistic and statistical methods in forecasting influenza-like illness. *J R Soc Interface* 2018; **15**(144).
9. Chinese Nataional Influenza Center. Influenza weekly report. http://www.chinaivdc.cn/cnic/en/ (accessed July 27 2020).
10. Center for Health Protection. Flu Express Volume 15 Number 5 (published on 8 February 2018). 2018. https://www.chp.gov.hk/files/pdf/fluexpress_week05_08_02_2018_eng.pdf (accessed April 3 2018).
11. Center for Health Protection. Influenza virus subtyping in 2018. 2018. https://www.chp.gov.hk/en/statistics/data/10/641/643/6781.html (accessed April 4 2018).
12. Chuang JH, Huang AS, Huang WT, et al. Nationwide surveillance of influenza during the pandemic (2009-10) and post-pandemic (2010-11) periods in Taiwan. *PLoS One* 2012; **7**(4): e36120.
13. Taiwan Centers for Disease Control. Taiwan Influenza Express. 2020. https://www.cdc.gov.tw/En/Category/MPage/Utv3lzlSnTK-t6inZrBZsw (accessed July 9 2020).
14. Korea Centers for Disease Control and Prevention. Weekly Sentinel Surveillance Report. 2020. http://www.cdc.go.kr/npt/biz/npp/portal/nppPblctDtaView.do?pblctDtaSeAt=2&pblctDtaSn=2165 (accessed July 9 2020).
15. World Health Organization (WHO), Global Influenza Programme, FluID (31 Dec 2021), https://www.who.int/teams/global-influenza-programme/surveillance-and-monitoring/fluid. 2021.
16. World Health Organization (WHO), Global Influenza Programme, FluNet (31 Dec 2021), https://www.who.int/tools/flunet. 2021.

17. IDWR Surveillance Data Table 2021 week 27 [retrieved on 2021 July 24]; Available from: https://www.niid.go.jp/niid/en/from-lab-e/from-idsc-e.html. 2021.
18. Sakamoto H, Ishikane M, Ueda P. Seasonal Influenza Activity During the SARS-CoV-2 Outbreak in Japan. *JAMA* 2020.
19. Kramer SC, Shaman J. Development and validation of influenza forecasting for 64 temperate and tropical countries. *PLoS Comput Biol* 2019; **15**(2): e1006742.
20. Public Health England - GOV.UK; Weekly national flu reports, (31 Dec 2021), https://www.gov.uk/government/collections/weekly-national-flu-reports. 2021.
21. Zhao H, Green H, Lackenby A, et al. A new laboratory-based surveillance system (Respiratory DataMart System) for influenza and other respiratory viruses in England: results and experience from 2009 to 2012. *Euro Surveill* 2014; **19**(3).
22. Centers for Disease Control and Prevention. National, Regional, and State Level Outpatient Illness and Viral Surveillance. https://gis.cdc.gov/grasp/fluview/fluportaldashboard.html (accessed July 27 2020).
23. Wu P, Tsang T, Wong J, et al. Suppressing COVID-19 transmission in Hong Kong: an observational study of the first four months. *09 June 2020, PREPRINT (Version 1) available at SSRN: https://paperssrncom/sol3/paperscfm?abstract_id=3627304* 2020.
24. Ali ST, et, al. figshare. Table S1. https://doi.org/10.6084/m9.figshare.18517028. 2022.
25. te Beest DE, van Boven M, Hooiveld M, van den Dool C, Wallinga J. Driving factors of influenza transmission in the Netherlands. *Am J Epidemiol* 2013; **178**(9): 1469-77.
26. Cowling BJ, Chan KH, Fang VJ, et al. Comparative epidemiology of pandemic and seasonal influenza A in households. *New England Journal of Medicine* 2010; **362**: 2175-84.
27. Ali ST, Kadi AS, Ferguson NM. Transmission dynamics of the 2009 influenza A (H1N1) pandemic in India: the impact of holiday-related school closure. *Epidemics* 2013; **5**(4): 157-63.
28. Cauchemez S, Bhattarai A, Marchbanks TL, et al. Role of social networks in shaping disease transmission during a community outbreak of 2009 H1N1 pandemic influenza. *Proc Natl Acad Sci U S A* 2011; **108**(7): 2825-30.
29. Cauchemez S, Ferguson NM, Wachtel C, et al. Closure of schools during an influenza pandemic. *Lancet Infect Dis* 2009; **9**(8): 473-81.
30. Cauchemez S, Valleron AJ, Boelle PY, Flahault A, Ferguson NM. Estimating the impact of school closure on influenza transmission from Sentinel data. *Nature* 2008; **452**(7188): 750-4.
31. Ferguson NM, Cummings DA, Fraser C, Cajka JC, Cooley PC, Burke DS. Strategies for mitigating an influenza pandemic. *Nature* 2006; **442**(7101): 448-52.
32. Ali ST, Wang L, Lau EHY, et al. Serial interval of SARS-CoV-2 was shortened over time by nonpharmaceutical interventions. *Science* 2020; **369**(6507): 1106-9.
33. Cowling BJ, Chan KH, Fang VJ, et al. Comparative epidemiology of pandemic and seasonal influenza A in households. *N Engl J Med* 2010; **362**(23): 2175-84.
34. te Beest DE, Wallinga J, Donker T, van Boven M. Estimating the generation interval of influenza A (H1N1) in a range of social settings. *Epidemiology* 2013; **24**(2): 244-50.
35. Carrat F, Vergu E, Ferguson NM, et al. Time lines of infection and disease in human influenza:

a review of volunteer challenge studies. *Am J Epidemiol* 2008; **167**(7): 775-85.
36. Young B, Sadarangani S, Jiang L, Wilder-Smith A, Chen MI. Duration of Influenza Vaccine Effectiveness: A Systematic Review, Meta-analysis, and Meta-regression of Test-Negative Design Case-Control Studies. *J Infect Dis* 2018; **217**(5): 731-41.
37. Anderson RM, May RM. Age-related changes in the rate of disease transmission: implications for the design of vaccination programmes. *J Hyg (Lond)* 1985; **94**(3): 365-436.
38. Osterholm MT, Kelley NS, Sommer A, Belongia EA. Efficacy and effectiveness of influenza vaccines: a systematic review and meta-analysis. *Lancet Infect Dis* 2012; **12**(1): 36-44.
39. Massad E, Coutinho FA. Vectorial capacity, basic reproduction number, force of infection and all that: formal notation to complete and adjust their classical concepts and equations. *Mem Inst Oswaldo Cruz* 2012; **107**(4): 564-7.
40. King AA, Nguyen D, Ionides EL. Statistical Inference for Partially Observed Markov Processes via the R Package pomp. *J Stat Softw* 2016; **69**(12).
41. He D, Ionides EL, King AA. Plug-and-play inference for disease dynamics: measles in large and small populations as a case study. *J R Soc Interface* 2010; **7**(43): 271-83.
42. King AA. POMP: statistical inference for partially-observed Markov processes. http://kingaa.github.io/pomp/; accessed on Mar, 2021.
43. Ionides EL, Nguyen D, Atchade Y, Stoev S, King AA. Inference for dynamic and latent variable models via iterated, perturbed Bayes maps. *Proc Natl Acad Sci U S A* 2015; **112**(3): 719-24.
44. Ionides EL, Breto C, Park J, Smith RA, King AA. Monte Carlo profile confidence intervals for dynamic systems. *J R Soc Interface* 2017; **14**(132).
45. Centre for Disease Control and prevention (CDC), Hong Kong SAR, Key Facts About Influenza (Flu), (9 June 2022), https://www.cdc.gov/flu/about/keyfacts.htm. 2022.
